# Effect of BET Missense Mutations on Bromodomain Function, Inhibitor Binding and Stability

**DOI:** 10.1371/journal.pone.0159180

**Published:** 2016-07-12

**Authors:** Laura Lori, Alessandra Pasquo, Clorinda Lori, Maria Petrosino, Roberta Chiaraluce, Cynthia Tallant, Stefan Knapp, Valerio Consalvi

**Affiliations:** 1 Department of Biochemical Sciences “A. Rossi Fanelli”, Sapienza University of Rome, Rome, Italy; 2 SSPT-BIOAG-BIOTEC ENEA Casaccia ENEA, Rome, Italy; 3 Nuffield Department of Clinical Medicine, Structural Genomics Consortium and Target Discovery Institute, University of Oxford, Oxford, United Kingdom; George Mason University, UNITED STATES

## Abstract

Lysine acetylation is an important epigenetic mark regulating gene transcription and chromatin structure. Acetylated lysine residues are specifically recognized by bromodomains, small protein interaction modules that read these modification in a sequence and acetylation dependent way regulating the recruitment of transcriptional regulators and chromatin remodelling enzymes to acetylated sites in chromatin. Recent studies revealed that bromodomains are highly druggable protein interaction domains resulting in the development of a large number of bromodomain inhibitors. BET bromodomain inhibitors received a lot of attention in the oncology field resulting in the rapid translation of early BET bromodomain inhibitors into clinical studies. Here we investigated the effects of mutations present as polymorphism or found in cancer on BET bromodomain function and stability and the influence of these mutants on inhibitor binding. We found that most BET missense mutations localize to peripheral residues in the two terminal helices. Crystal structures showed that the three dimensional structure is not compromised by these mutations but mutations located in close proximity to the acetyl-lysine binding site modulate acetyl-lysine and inhibitor binding. Most mutations affect significantly protein stability and tertiary structure in solution, suggesting new interactions and an alternative network of protein-protein interconnection as a consequence of single amino acid substitution. To our knowledge this is the first report studying the effect of mutations on bromodomain function and inhibitor binding.

## Introduction

Epigenetics has been defined as heritable changes in phenotype that are the consequence of changes in DNA sequence but are due to differences in the pattern of post-translational modification present in histone, other nuclear proteins and in DNA [1]. Changes in post-translational modifications also called epigenetic marks is a principal mechanism regulating chromatin structure and gene transcription and dysregulation of epigenetic marks has been linked to the development of a large diversity of diseases. Acetylation of lysine residues (Kac) is one of the most frequently occurring post-translational modifications which controls a vast array of diverse cellular functions. Dysregulation of acetylation levels has been associated with the development of many diseases in particular to cancer and enzymes regulating acetylation have emerged as interesting targets for drug discovery [2–4]. Acetylation levels are reversibly maintained by a group of enzymes, the histone acetyl-transferases and histone deacetylases that “write” and “erase” acetylation marks on histones [5]. Acetylation sites in proteins are specifically recognized by small helical interaction modules called Bromodomains (BRDs).

The relevant importance of BRDs in drug design is highlighted in recent studies that report BRDs as a target site for the development of new cancer drugs [3, 6–9]. Inhibitors that specifically target the BET (Bromo and Extra Terminal) proteins selectively interfered with gene expression that mediated cellular growth and evasion of apoptosis in cancer [10–12]. The studies of these inhibitors have suggested that inhibition of BRDs may have several potential clinical applications [3, 13]. BET proteins (ubiquitously expressed BRD2, BRD3, BRD4 and testis-specific BRDT) belong to the subfamily II of BRDs, sharing a common architecture comprising two N-terminal BRDs, domain 1 and domain 2, that exhibit high level of sequence conservation as well as an extra terminal domain and a more divergent C-terminal recruitment domain.

Despite their low sequence identity, all BRDs share a conserved fold comprising a left-handed bundle of four alpha helices, connected with a characteristic hydrophobic cleft between two conserved loops [14]. This binding site specifically recognizes ε-aminoacetyl groups of nucleosomal histone. The first bromodomains of BETs have a preference binding to di-acetylated Kac present in histone H4. The binding mode of the acetyl-lysine interaction is highly conserved comprising an anchoring hydrogen bond to a conserved asparagine residue present in most BRDs as well as a water mediated hydrogen bond to a conserved tyrosine residue [15].

BRD4 and BRD2 have crucial roles in cell cycle control [16, 17]. BRD2 and BRD4 remain bound to mitotic chromatin [18]; this property has been suggested to be important for the maintenance of epigenetic memory during cell division [19, 20]. Constitutive over-expression of BRD2 in B-cells of transgenic mice results in the development of B-cell lymphoma and leukaemia [21]. Gene rearrangements of BRD3 and BRD4 with a testis specific protein called NUT (Nuclear protein in Testis) have been detected in aggressive carcinoma [22–24]. BRD3 expression is induced in activated lymphocytes and it is highly expressed in undifferentiated embryonic stem cells, whereas expression levels are reduced upon endothelial differentiation [25]. Down regulated expression or loss of BRD3 has been detected in biopsies of nasopharyngeal carcinomas [26] and altered expression levels have been found in bladder cancer [25].

Several mutations in BRDs have been identified in humans and they may play an important role in several diseases but the functional consequences of the recorded mutations has not been studied. These variants are nonsynonymous single nucleotide polymorphisms (nsSNPs), single nucleotide variations occurring in the coding region and leading to a polypeptide sequence with amino acid substitutions. A number of investigations have addressed the effect of nsSNPs on protein stability, protein-protein interactions and protein functions for several other protein families [27, 28]. Indeed, large-scale computational studies utilizing structural information indicate that many nsSNPs may affect protein stability by either increasing or decreasing protein stability. However, only a detailed experimental analysis can unequivocally reveal the effect of the missense mutation on protein function [29, 30].

Thus, a detailed characterization of BET bromodomain variants found in cancer will be important to evaluate the role of these sequence alteration affecting bromodomain function and potentially resistance to emerging treatment strategies. Indeed, recent reports already identified mechanisms by which cancer cells may evade treatment by BET bromodomain inhibitors [31, 32]. We therefore report here the effect of BET bromodomain mutations found in the cancer patients and annotated in the COSMIC database (http://cancer.sanger.ac.uk/cosmic) [33] on bromodomain structure, the ability of BET bromodomains to bind acetylated target sequences as well as inhibitors and the consequences of these mutations on the conformational stability of the bromodomain fold.

## Results

### BET variants in cancer

**Fig 1:**
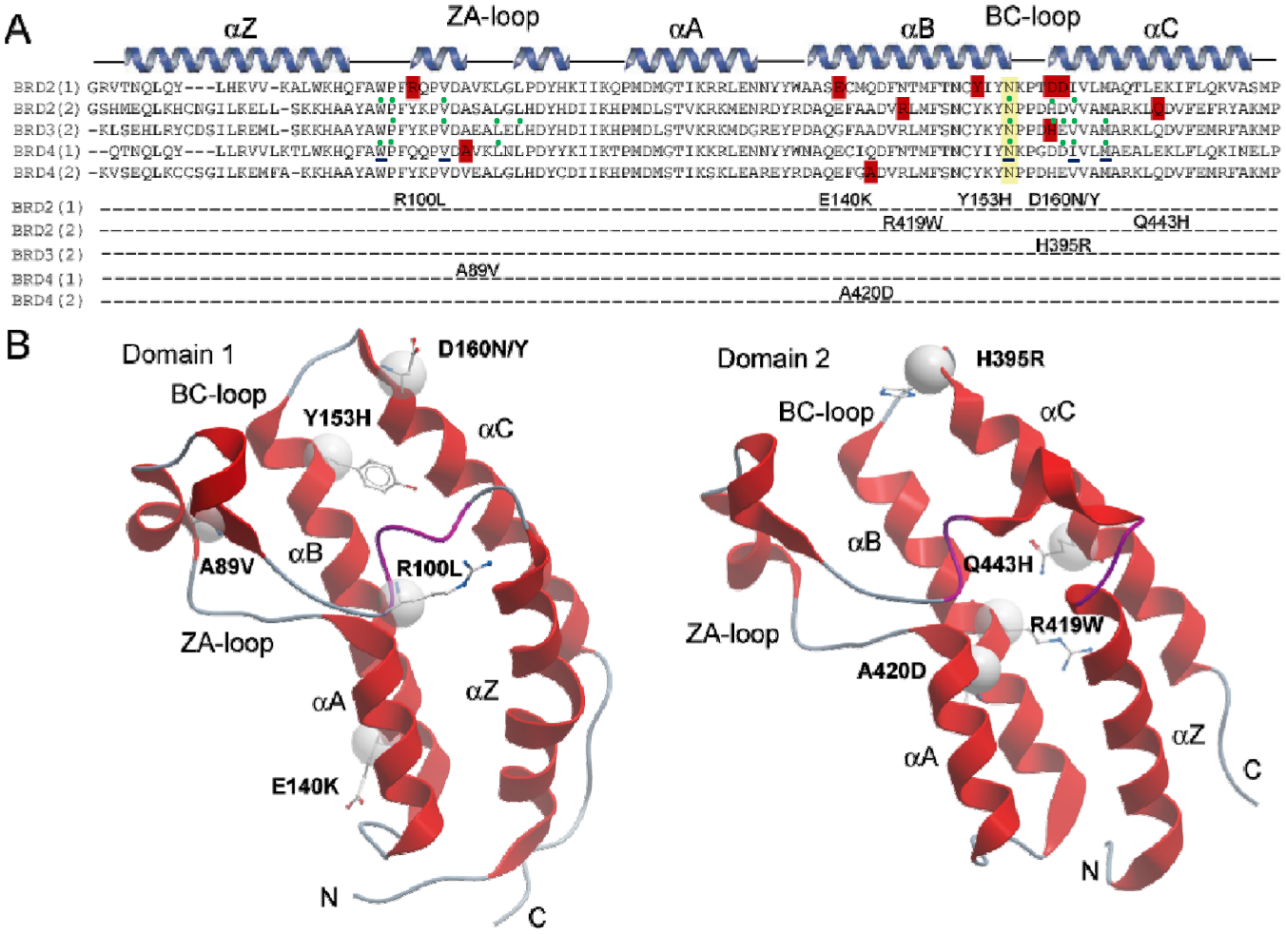
Alignment of BET bromodomain mutants. (A) Secondary structure elements are shown at the top of the sequence alignment. Mutated residues are highlighted in red and studied mutations are listed. The conserved asparagine (N391in BRD3(2) numbering) is highlighted in yellow. The green dots represent the residues involved in binding with inhibitor JQ1 (PDB ID: 3ONI, 3S92, 3MXF). The residues underlined in blue are involved in PFI-1binding (PDB ID: 4E96). (B) Location of the mutations. Shown are the first (left) and second bromodomain of BRD2. The mutated residues are highlighted in ball and stick and the position of Cα atoms are shown as a sphere. The main structural elements are labelled.

Mutants that map to BET bromodomains were mined from the COSMIC database (http://cancer.sanger.ac.uk/cosmic) [33] and were mainly identified BET variants, present in cancer of the large intestine and lung or in hematopoietic malignancies. Most of the nsSNPS found in the COSMIC database were present in BRD2. We identified seven mutants in BRD2 in the sequence spanning both bromodomains (D160N, D160Y, Y153H, E140K, R100L, Q443H, R419W), one in the second bromodomain of BRD3 (2) (H395R) and two mutations in the first and second bromodomain of BRD4 (A89V, A420D) (Fig 1A). Most mutants were located on the two terminal helices αB and αC, some of them in the proximity of the acetyl-lysine binding site and in the loop regions (e.g. A89V in BRD4(1) and R100L in BRD2(1) are located in the ZA loop) but no sequence variants were found in the highly sequence diverse αA helices. The mutated residues present in the αB helix, namely E140K and Y153H in BRD2(1), R419W in BRD2(2), A420D in BRD4(2), do not alter the conserved sequence motif ϕxxDϕxxϕϕxNϕxxY (ϕ is a hydrophobic residue) that precedes the conserved arginine essential for Kac binding in BET proteins [14] but the tyrosine residue Y153, mutated in BRD2(1), is highly conserved in bromodomains suggesting a role in stabilizing the bromodomain fold. The residues D160 in BRD2(1) and H395 in BRD3(2), mutated in D160N, D160Y and H395R, respectively are located at the C-terminus of the BC loop, a region important for binding of Kac peptides and inhibitors (Fig 1A). The residue D161, located in the Kac binding region, was also mutated into D161N and D161Y to test the effect of this amino acid replacement in a region important for acetylated peptides binding. The location of the identified mutants mapped onto the structures of the first and second bromodomains is shown in Fig 1B [14]. All the mutations studied involve surface exposed residues and do not affect conserved amino acids, with the exception of Y153, a tyrosine residue present in all the sequence alignment in the family II BRDs (Fig 1). Hence, the consequences of the mutations were not obvious and we generated recombinant protein for each of the identified mutants using site directed mutagenesis and available bacterial expression systems [14].

### Effects of BET mutants on structure

**Fig 2:**
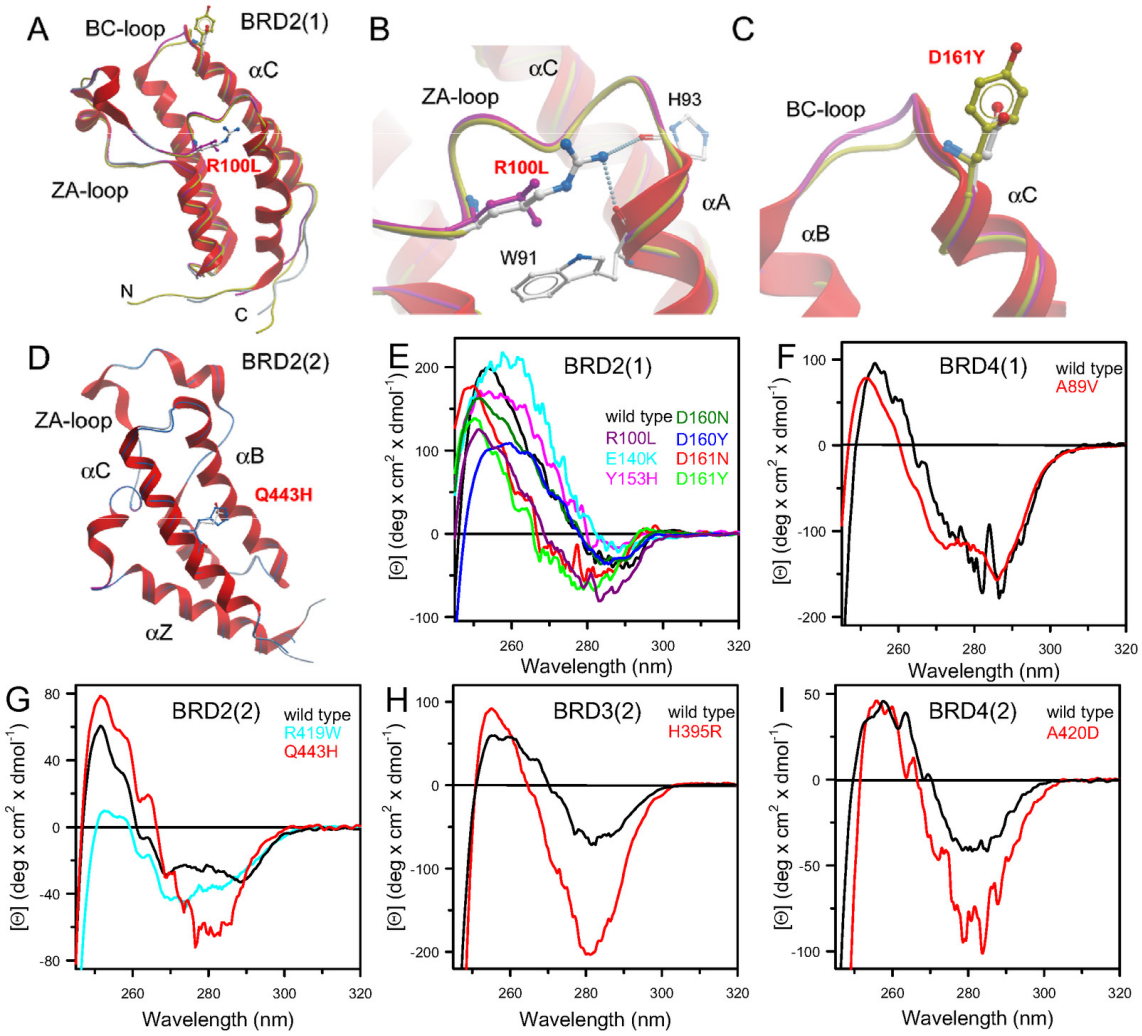
Structure of BET mutants and tertiary structure of mutants in solution. (A) Superimposition of wild type BRD2(1) shown as a ribbon diagram with the mutants BRD2(1) R100L and D161Y shown as protein worm in green and magenta, respectively. The mutated residues are shown in ball and stick representation and main structural elements are labelled. (B) Details of interactions formed by R100 in the wild type compared to the mutated residue. (C) Detailed view of BRD2(1) wild type and D161Y. (D) Superimposition of BRD2(2) shown as a ribbon diagram and the mutant BRD2(2) Q443H shown as protein worm in blue. (E) Comparison of the near UV CD spectra of wild type BRD2(1) and all generated mutants. (F) Comparison of the near UV CD spectra of wild type BRD4(1) and the mutants BRD4(1) A89V. (G) Comparison of the near UV CD spectra of wild type BRD2(2) and the mutants BRD2(2) R419W and Q443H. (H) Comparison of the near UV CD spectra of wild type BRD3(2) and the mutants BRD3(2) H395R. (I) Comparison of the near UV CD spectra of wild type BRD4(2) and the mutants BRD4(2) A420D. Near-UV CD spectra were recorded at 20°C in a 1.0-cm quartz cuvette in 20 mM Tris/HCl, pH 7.5 containing 0.20 M NaCl and 2.00 mM DTT, as described in Materials and Methods.

In order to obtain insight on changes in three dimensional structure as well as local interaction of the mutated sidechains we solved crystal structures of some of the generated mutants (Fig 2). All structures (BRD2(1)R100L, BRD2(1)Y153H, BRD2(1)D161Y, BRD2(2)Q443H, BRD3(2)H395R) were refined to high resolution maintaining favourable geometry (S1 Table). The three dimension structure of the mutants was found to be highly conserved showing only local structural alterations (Fig 2A). In the BRD2(1) mutant R100L the hydrogen bonds with backbone residues in helix αA were lost but this did not result in significant structural changes (Fig 2B). Mutations at BRD2(1) D161 as well as BRD2(2) Q443H (Fig 1) did also not result in any significant structural rearrangements in the refined crystal structures (Fig 2C and 2D).

### Effects of BET mutants on conformation in solution

The conformation in solution of all mutants was studied spectroscopically using CD and fluorescence spectroscopy. The near-UV CD spectra of wild type bromodomains represent the spectral contributions of all aromatic residues and was characterized by a strong negative peak centred at around 280 nm and a positive one around 260 nm, accompanied, for BRD4(1) and BRD2(2), by fine structure features in the region of 290 nm (Fig 2E–2I). Notably, the significant differences observed in the near UV CD spectra of all the mutants indicated that their tertiary structure arrangements are different from that of the corresponding wild type proteins, as also suggested by differences observed in the intrinsic fluorescence spectra (S1 Fig). The intrinsic fluorescence spectra of most of the variants differed from the corresponding wild type proteins mainly in intensity, which was either significantly decreased or enhanced. As expected, based on differences in aromatic residue content, the maximal fluorescence emission wavelength of BRD2(2) R419W was red shifted with respect to that of the wild type protein (S1C Fig). These results pointed to local rearrangements of tertiary interactions for most of the BRDs variants in solution.

**Fig 3:**
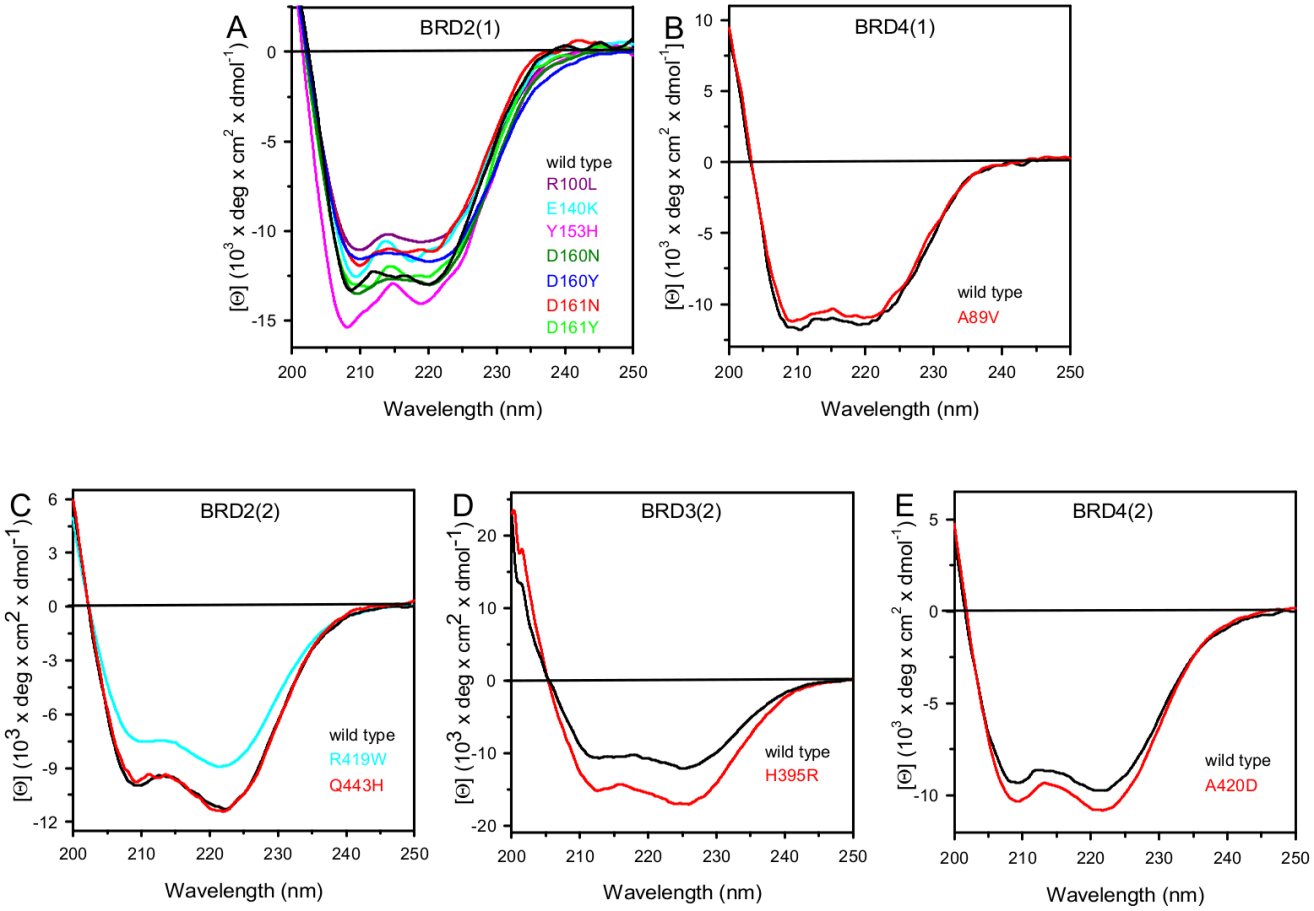
Far-UV CD spectra of wild type bromodomains and mutants. Far-UV CD spectra were recorded at 20°C in a 0.1-cm quartz cuvette in 20 mM Tris/HCl, pH 7.5 containing 0.20 M NaCl and 0.40 mM DTT, as described in Materials and Methods. Wild type spectra are shown as black solid lines and mutants are coloured as indicated in the figure.

The far-UV CD spectrum of all mutants indicated some differences in secondary structure content probably due to differences in dynamic fluctuation in solution (Fig 3). The 222/208 ellipticity ratio, which is indicative of interhelical contacts present in helix bundle and coiled coil structures as expected in the conserved bromodomain fold, differed in the various wild type bromodomains and corresponded to 0.97, 0.98, 1.06, 1.16 and 2.15 in BRD2(1), BRD4(1), BRD4(2), BRD2(2) and BRD3(2), respectively. The 222/208 ellipticity ratio is generally used to distinguish between coiled coil helices (≥ 1.0) and non-interacting helices (0.8–0.9) [34, 35]. This ratio was unchanged for all the variants, except for the BRD2(1) mutants Y153H and E140K that show a 222/208 ellipticity ratio below 1.0, corresponding to 0.86 and 0.91, respectively (Fig 3A). The decrease in the 222/208 ellipticity ratio observed for these BRD2(1) variants suggested that a consequence of the amino acid substitutions was a perturbation of interhelical contacts that resulted in structural differences in solution, despite the fact that Y153 and E140 are not involved in any direct interactions with another helix and are solvent exposed (Fig 1B).

All in all we conclude that despite the high structural conservation of mutants in crystal structures the studied bromodomain mutants showed detectable changes of their spectroscopic properties in solution suggesting differences in three dimension structures and the dynamic properties of these mutants. Proteins are conformationally constrained in crystals and the studied bromodomain mutations may therefore alter domain plasticity and structural properties that are only apparent in solution.

### Effects of BET mutants on acetyl-lysine peptide and inhibitor binding

**Fig 4:**
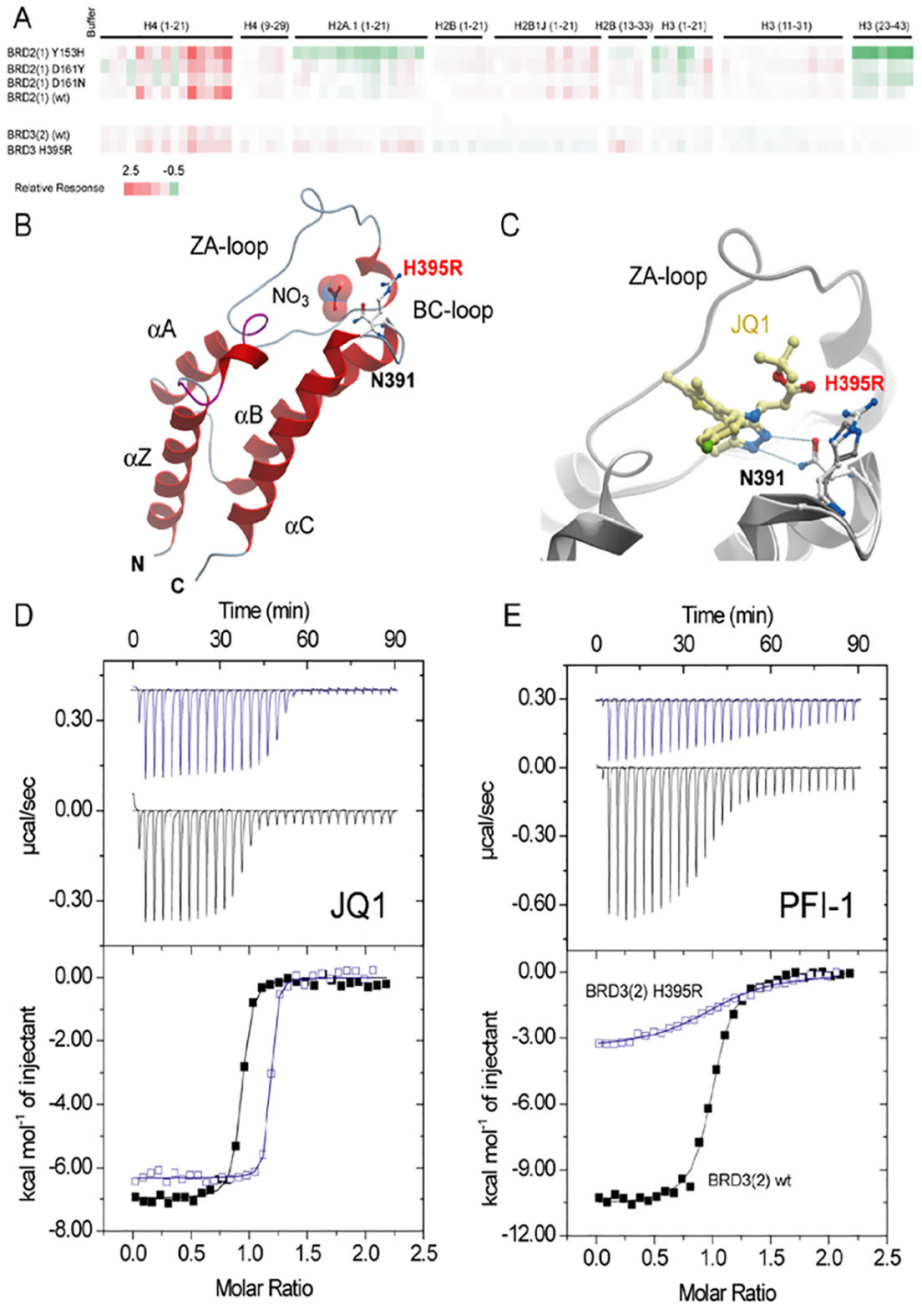
Binding of BET bromodomain mutants to acetylated histone peptides and inhibitors. (A) BLI study showing the interaction of some mutants with acetylated histone peptides. Shown is the maximum response at a protein concentration of 20 μM after subtraction of non-acetylated reference peptides using a colour code as indicated in the figure capture. (B) Structure of BRD3(2) mutant H395R. The mutated residues are highlighted. A nitrate molecule, present in the crystallization solution occupied the acetyl-lysine binding site in BRD3(2) H395R. The conserved asparagine (N391) formed canonical hydrogen bonds with the nitrated ion. (C) Superimposition of the wild type JQ1 complex with BRD3(2) H395R. (D) ITC experiments measuring the interaction of the panBET inhibitor JQ1 with wild type BRD3(2) (black curve) and BRD3(2) H395R (blue curve). Shown are raw titration heats (top panel) as well as normalized binding enthalpies as a function of ligand/protein ratio (lower panel). The best fit to a single binding site model is shown as solid lines. (E) ITC experiments showing the binding of the pan-BET inhibitor PFI-1 with BRD3(2) and the H395R mutant. A significant reduction in binding enthalpy and binding affinity is evident. Data on all ITC titrations are summarized in S2 Table.

Some of the BET mutants identified in cancer, are located in close proximity to the acetyl-lysine binding site and we were therefore interested whether these mutants compromised the ability of the bromodomain to bind acetylated histone peptides as well as BET inhibitors. To address the first question we used biolayer interference (BLI), a technology that measures differences in refractive index in a similar way as the widely used surface plasmon resonance technology, and a library of biotin labelled acetylated histone peptides. Using this peptide library we measured the BLI response at 20 μM protein concentration on tips with immobilized acetylated and non-acetylated control peptides (Fig 4A). As expected, the first bromodomain of BRD2(1) interacted strongly with polyacetylated peptides of histone H4 [14]. This interaction was weakened by mutation in a region important for binding of Kac peptides (Fig 1A), such as D161 (Fig 4A). Second bromodomains in BETs show much weaker interaction with histones and other nuclear proteins that have been discussed as potential targets [36]. Indeed, we confirmed these reports and found only weak interaction with histones peptides that were present in our peptide library for BRD3(2) (Fig 4A).

For studying the effects of the mutations on binding of BET inhibitors, we used two structurally diverse panBET inhibitors: the thienodiazepine JQ1 [11] as well as the quinazolinone PFI-1 [14, 37, 38]. Binding of JQ1 was only modestly affected by all mutations (S2 Table and Fig 4D). Notably, the *K*_d_ for JQ1 was slightly increased for A420D, D161N and Y153H and slightly decreased for all the other variants. A different trend is observed for PFI-1: in this case the *K*_d_ was slightly decreased for D161Y and A420D and was increased for all the other variants. The largest differences were observed for mutants of BRD3(2). H395R mutant and wild type protein with both the inhibitors JQ1 and PFI-1; this variant showed a 3-fold lower *K*_d_ for JQ1 and about 9-fold higher *K*_d_ for PFI-1(Fig 4E). Comparison with the crystal structure of wild type BRD3(2) with JQ1 and the structure of BRD3(2) H395R suggested that the arginine residue introduced by this mutant may form a more favourable hydrogen bond with the carbonyl oxygen of the ester link (Fig 4B and 4C). All titration experiments are compiled in S2 Table.

### Effects of BET mutants on BRD stability

The thermal and thermodynamic stability of the wild type BRD2(1), BRD4(1) BRD2(2), BRD4(2) and BRD3(2) were compared with the generated BET variants (Table 1).

**Table 1:** Melting temperatures and thermodynamic parameters for urea-induced unfolding equilibrium of BRDs wild type and mutants measured by far-UV CD and fluorescence spectroscopy.

	Tm (°C)	Δ*G*^H^_2_^O^ (kcal/mol)		*m* (kcal/mol/M)		[Urea]_0.5_ (M)	
		CD ([Θ]_222_)	Fluorescence	CD ([Θ]_222_)	Fluorescence	CD ([Θ]_222_)	Fluorescence
**BRD2(1)**							
Wild type	54.8	12.88 ± 0.93	12.91 ± 0.99	2.40 ± 0.17	2.23 ± 0.17	5.36	5.79
Y153H	47.8	6.39 ± 0.34	7.60 ± 0.61	1.49 ± 0.08	1.60 ± 0.13	4.30	4.75
E140K	44.0	9.40 ± 0.70	8.38 ± 0.63	2.16 ± 0.16	1.85 ± 0.14	4.35	4.54
R100L	49.0	7.66 ±0.87	∆*G*_1_ = 8.20 ∆*G*_2_ = 10.10	1.46 ± 0.16	m_1_ = 3.16 ± 0.66 m_2_ = 1.92 ± 0.14	5.42	2.47 ± 0.05 5.35 ± 0.02
D160N	49.0	8.78 ± 0.70	10.58 ± 0.82	2.03 ± 0.16	2.52 ± 0.19	4.32	4.20
D160Y	45.0	8.86 ± 0.59	8.71 ± 0.58	1.82 ± 0.12	1.80 ± 0.12	4.85	4.83
D161N	53.5	10.67 ± 0.91	9.89 ± 0.68	2.11 ± 0.33	2.09 ± 0.18	5.07	5.31
D161Y	50.0	9.50 ± 0.36	8.25 ± 0.47	1.71± 0.06	1.46 ± 0.08	5.55	5.63
**BRD4(1)**							
Wild type	52.9	8.82 ± 0.49	11.39 ± 0.97	1.27 ± 0.07	1.56 ± 0.13	6.96	7.32
A89V	53.0	9.81 ± 0.49	10.60 ± 0.83	1.46 ± 0.07	1.56 ± 0.12	6.70	6.80
**BRD2(2)**							
Wild type	53.0	7.97 ± 0.53	6.60 ± 0.39	1.50 ± 0.10	1.36 ± 0.08	5.29	4.86
Q443H	50.0	8.86 ±0.47	8.28 ± 0.40	1.77 ± 0.09	1.77 ± 0.09	5.01	4.68
R419W	45.0	7.95 ± 0.42	6.00 ± 0.36	1.72 ± 0.09	1.18 ± 0.07	4.63	5.10
**BRD3(2)**							
Wild type	51.6	7.14 ± 0.63	6.68 ± 0.52	1.41 ± 0.12	1.51 ± 0.11	5.06	4.44
H395R	49.0	9.17 ± 0.63	7.80 ± 0.55	2.02 ± 0.14	1.90 ± 0.13	4.53	4.10
**BRD4(2)**							
Wild type	54.0	8.66 ± 0.47	4.72 ± 0.23	1.59 ± 0.08	0.95 ± 0.05	5.43	4.95
A420D	51.0	9.10 ± 0.49	9.65 ± 0.53	1.75 ± 0.09	1.87 ± 0.10	5.20	5.15

The temperature-induced changes were followed by monitoring the ellipticity at 222 nm. The Tm values were calculated by taking the first derivative of the ellipticity at 222 nm with respect to temperature. Urea-induced unfolding equilibrium data were measured at 10°C in 20 mM Tris/HCl, pH 7.5, containing 0.2 M NaCl and 200 μM DTT by monitoring ellipticity at 222 nm [Θ_222_] and intrinsic fluorescence emission. Δ*G*^H^_2_^O^ and *m* values were obtained from Eq 3; [Urea]_0.5_ was calculated from Eq 2. Intrinsic fluorescence emission data of BRD2(1) R100L were fitted to Eq 5. Data are reported as the mean ± SE of the fit.

It is noteworthy that all the natural variants studied, with the exception of BRD4(1) A89V, showed a significant decrease in the melting temperature (Tm) ranging from 1.3°C for BRD2(1) D161N to 10.0°C for BRD2(1) E140K, when compared to the wild type proteins. The thermal stability of BRDs wild type and variants was investigated by continuously monitoring the ellipticity changes at 222 nm between 20 and 80°C. The observed thermal unfolding occured in an apparent two-state cooperative transition for all BRDs wild type and variants (S2 and S3 Figs). The midpoint of the unfolding, Tm, was calculated by plotting the first derivative of the molar ellipticity at 222 nm, where the main amplitude was observed, as a function of temperature (S2A and S2B Fig, inset; S3A–S3C Fig, inset). It is noteworthy that for all the BRDs variants, with the exception of BRD2(2) variants, the amplitudes of the ellipticity changes at 222 nm, i.e. the difference between the ellipticity measured at the end (80°C) and that at the beginning (20°C) of the thermal transition, were different from those measured for the wild type (S2C, S2D and S3D–S3F Figs). In particular, the amplitude of the ellipticity changes at 222 nm increased for all BRD2(1) variants, with the exception of D161N (S2C Fig), ranging from 1.3-fold for E140K, R100L, and D161Y, to 1.4-fold for D160N and D160Y and to 1.5-fold for Y153H. For the variants BRD3(2) H395R and BRD4(2) A420D a 1.6 and 1.2-fold increase of thermal transition amplitude is observed. These results point to a larger extent of loss of secondary structure upon thermal unfolding when compared to the wild type protein.

The lower Tm values and the higher loss of secondary structure elements upon thermal unfolding suggest that the point mutations induce a remarkable destabilization of the native state of BRDs. The temperature-induced ellipticity changes for all BRDs wild type and mutants were coincident with the heat-induced increase of the photomultiplier tube voltage (data not shown) suggesting that the temperature-induced unfolding is accompanied by protein aggregation [39]. Aggregation occurred also when thermal scans were performed at a lower heating rate with a low-temperature shifts of the apparent Tm; the differences between the apparent Tm of wild type and variants were the same as those measured at higher heating rate (data not shown). The observed transitions were irreversible as indicated by the spectra measured at the end of the cooling phase that differ from those of the native proteins measured at the beginning of the thermal transitions (data not shown).

The thermodynamic stability was studied by urea-induced equilibrium unfolding. BRD2(1), BRD4(1) BRD2(2), BRD4(2) and BRD3(2) wild type and variants reversibly unfold in urea at 10°C. The effect of increasing urea concentrations (0–8 M) on the structure of BRDs variants was analyzed by far-UV CD (S4A, S4B and S5A–S5C Figs) and fluorescence spectroscopy (S4C, S4D and S5D–S5F Figs) and compared to the effect exerted on the corresponding wild type. The same samples used to monitor the far-UV CD changes during the unfolding transition were used to monitor fluorescence emission changes, to allow a direct comparison of the data. The urea-induced changes in 222 nm ellipticity of all the BRDs wild type and mutants showed a sigmoidal dependence on denaturant concentration and follow an apparent two-state transition without any detectable intermediate (S4A, S4B and S5A–S5C Figs). Incubation of BRDs wild type and variants at increasing urea concentrations resulted in a progressive change of the intrinsic fluorescence emission intensity and a red-shift of the maximal emission wavelength from 345 nm, in the absence of denaturant, to about 366 nm, in 8 M urea (data not shown). Determination of the red-shift of the intrinsic fluorescence emission was obtained by calculating the intensity averaged emission wavelength, $\bar{\lambda }$, at increasing urea concentration (S4C, S4D, S4A, S4B and S5D–S5F Figs). This parameter is an integral measurement, negligibly influenced by the noise, and reflects changes in both the shape and the position of the emission spectrum. The urea-induced changes in $\bar{\lambda }$ of all the mutants are similar to that of the wild type proteins, show a sigmoidal dependence on urea concentration and follow an apparent two-state transition without any detectable intermediate, except for the variant of BRD2(1) R100L.

The thermodynamic parameters obtained from the analysis of the far-UV CD and fluorescence changes transitions are reported in Table 1. The difference between the free energy of urea-induced unfolding, Δ*G*^H^_2_^O^, of the variants and that of the wild type indicates a decrease in thermodynamic stability of 2–6 kcal/mol for BRD2(1) variants and an increase of about 2 kcal/mol for BRD3(2) mutant H395R. A minor increase in Δ*G*^H^_2_^O^ (about 1 kcal/mol) was observed for all the other BRDs variants (Table 1). The decrease in Δ*G* values measured monitoring the far-UV CD changes of the BRD2(1) variants Y153H, R100L, D160Y and D161Y may be mainly referred to the lower *m* values, with respect to the wild type proteins. An increase in both Δ*G* and *m* values was observed only for the BRD3(2) mutant H395R. The changes in *m* values may indicate differences in the solvent exposed surface area upon unfolding between the variants and the wild type: decrease in *m* values is usually referred to a decrease in the solvent-exposed surface area upon unfolding. This is frequently ascribed to an increase in the compactness of the residual structure in the non-native state ensemble, rather than to an increase of the accessible surface area of the native state [40–42]. A decrease in *m* value upon single mutation has been also referred, in some cases, to the population of a third intermediate state during chemical unfolding [43]; this is the case for R100L unfolding where an intermediate has been detected by monitoring the fluorescence changes (S4C Fig, inset). In 8.0 M urea, the ellipticity at 222 nm and the maximal fluorescence emission wavelength of the BRDs variants are comparable to those of the corresponding wild type proteins (data not shown) and did not indicate any increase in the structure of the non-native state to support the large decrease in *m* value observed for the BRD2(1) variants Y153H, R100L, D160Y and D161Y. In native conditions, the spectral properties of most of the variants point to tertiary structure arrangements different from those of the wild type and, for BRD2(1) E140K and Y153H variants (Fig 2), the 222/208 nm ratio < 1.0 (Fig 3A) may suggest a less compact native state.

The values of the thermodynamic parameters obtained from far-UV CD were comparable to those determined from fluorescence data for all the BRDs wild type, with the exception for BRD4(1) and BRD4(2) (Table 1); a non-coincidence of the thermodynamic parameters obtained by the two spectroscopic probes was also observed for the BRD2(2) variant R419W (Table 1). The lack of coincidence of the thermodynamic parameters determined using different spectral probes is generally considered as an indication that the unfolding does not follow a simple two-state process and suggests the presence of an undetected intermediate in the unfolding pathway. However, the *m* values obtained from the analysis of the far-UV CD and fluorescence unfolding transitions were within the range of those expected for a monomeric protein of about 115 amino acid residues denatured in urea [44]. Indeed, the presence of a folding intermediate was evident for the BRD2(1) R100L whose reversible urea unfolding transition monitored by the changes in fluorescence intensity averaged emission wavelength $\bar{\lambda }$ (S4C Fig, inset) was fitted as a three-state unfolding process, according to Eq 5 (Table 1).

## Discussion

Bromodomains (BRDs) dysfunction has been linked to the development of several diseases [4]. In these regards, BRDs have recently emerged as interesting targets for the development of specific protein interaction inhibitors [3]. The object of this study were three of the four BET proteins BRD2, BRD3 and BRD4 and to our knowledge, this is the first spectroscopic characterization in solution of human BRDs variants found in cancer. Wild type and variants of BRD2(1), BRD4(1), BRD2(2), BRD3(2) and BRD4(2), were studied in order to investigate the effect of amino acid substitutions on their structure in solution and on their thermal and thermodynamic stability and interactions with peptides and inhibitors (Figs 1 and 2, S6 Fig). The structures of some of the variants that showed the most significant changes in inhibitor binding were determined by X-ray crystallography (Fig 2).

The single amino acid substitutions significantly affect the tertiary interactions of most of the BRDs variants studied, as indicated by the differences in their near UV CD fine structure and/or in the ellipticity amplitude, when compared to the corresponding wild type proteins. Due to the usually surface exposed location of the mutated residues, the consequences of the mutations were not obvious, however most of the amino acid substitutions in BRDs variants involve changes in the charge of solvent exposed residues, that may alter protein stability. Notably, the amino acid substitutions in most of the variants suggest an increase in side chain flexibility for the mutated residue [45] that may be responsible for local changes in protein dynamics. The changes in tertiary arrangements of most of the variants were also evident from their intrinsic fluorescence spectra that showed differences in relative intensity and/or in the maximum emission wavelength. Only the mutation of Ala89 to Val did not significantly affect the tertiary contacts of BRD4(1), as judged by a comparison of its spectral properties and of its stability with those of the wild type. A loosening of the native tertiary structure was suggested also by the changes in the far UV CD spectra of some BRDs variants, particularly those of BRD2(1) Y153H and E140K for which the 222/208 ellipticity ratio below 1.0 points to a weakening of the interhelical contacts [34, 35]. Consequently the thermal and thermodynamic stability of these two variants were significantly decreased, as expected from the role of the conserved Y153 residue, located at the end of the αB helix, in stabilizing the bromodomain fold [14]. On the other hand, the substitution of the negatively charged glutamate with a positively charged lysine may induce local perturbations responsible for the observed destabilization, despite the lack of interaction of the solvent exposed side chain of E140 in the crystal structure of the wild type protein.

The thermal and thermodynamic stability of the BRD2(1) variants Y153H, E140K, R100L, D160N, D160Y and D161Y were remarkably lower than the corresponding wild type: the Tm values were at least 5 degrees below that of the wild type and the Δ*G* values are more than 3 kcal/mol lower than those of the wild type proteins (S3 Table). Amino acid substitutions in BRD2(1) variants involved changes in the charge of solvent exposed residues which would be expected to have a significant impact on protein stability. In general, the naturally occurring point mutations in BRD2(1) variants induced a remarkable destabilization of the native state, as suggested by the significant loss of secondary structure elements upon thermal unfolding and by the decrease in Tm and/or in Δ*G* values, e.g. in the mutant of the conserved Y153. However, for the other BRDs variants, differently from what observed for those of BRD2(1), the perturbation of tertiary contacts were not always accompanied by changes in both thermal and thermodynamic stability. The 8°C decrease in thermal stability of BRD2(2) R419W variant was not paralleled by a drop of its thermodynamic stability measured by urea induced-unfolding. Mutation of a charged arginine residue, solvent exposed, into an aromatic and more flexible tryptophan [45] may induce tertiary changes and lead to a protein with different hydrophobic interactions and, probably, more prone to thermal denaturation. On the other hand, for the BRD3(2) variant H395R the Tm is 2.6°C lower than that of the wild type, whereas the unfolding Δ*G* is even higher than that of the wild type, due to the higher *m* value of this variant (Table 1). Interestingly, both the variants BRD2(2) R419W and BRD3(2) H395R displayed significant differences in the overall far-UV CD ellipticity and in the tertiary arrangements, as indicated by the difference of their intrinsic fluorescence emission and near UV CD spectra with respect to the wild type, despite the fact that interhelical contacts in these variants were unaffected by the mutation, as suggested by the 222/208 ellipticity ratio similar to that of the wild type. In conclusion, the naturally occurring mutations in BRDs, caused by nsSNPs and found in cancer result in proteins with significantly altered physico-chemical properties, such as alteration of native tertiary contacts, loosening of the interhelical contacts or changes in thermal and/or thermodynamic stability [46]. In conclusion, despite the high structural conservation in crystal structures, the BRDs variants showed differences in spectral properties in solution that may suggest local structural changes and modifications of their dynamic properties.

The binding studies revealed that the BRD2(1) variants, namely Y153H, D161Y and D161N and BRD3(2) H395R showed significant differences compared to the wild-type proteins. The observed differences in inhibitor binding (S2 Table) may be referred to the fact that the mutated residues are located in close proximity of the BC loop, a region important for binding of Kac peptides and inhibitors (Fig 1A). Also the interactions with the acetylated histones peptides of H2A, H2B, H3 and H4, although preserved, showed some differences indicating that the mutations affect the binding interactions with the natural substrates. Interestingly, the superimposition of the X-ray structures of some of the variants with those of the wild type, revealed that the mutations did not cause overall misfolding of the structure (Figs 2 and 4). Minor structural changes were observed in BRD3(2) H395R where the amino acid substitution resulted in a new hydrogen bond and affect interactions in the ligand binding site, as indicated by the different inhibitor binding affinity. Notably the remarkable changes in the interactions with histone recognition sequences and inhibitors, e.g. in the H395 mutant, were not accompanied by significant changes in the thermal and or thermodynamic stability of this variant (Fig 4 and S3 Table). A close relationship between the stability and the inhibitor binding data can be established only for Y153H which showed the largest differences in stability accompanied by a significant decrease in binding affinity for JQ1 and PFI-1.

Taken altogether, our results indicate that the mutants of BRDs found in cancer tissues did not alter significantly the overall folding. However, the significant alteration of the tertiary contacts observed in solution and the notable decrease in protein stability suggested an increase in conformational flexibility. All the mutated residues were solvent exposed, therefore they are not supposed to alter the global folding; however, a mutation of a residue on the surface may result in new and unknown interactions, thus the variants may acquire a new pattern of interactions and establish a novel and alternative network of protein-protein interconnection [47, 48]. This may be particularly important in BRDs that are physiologically embedded in multi-domain proteins and multi-subunit complexes.

Apart from PFI-1 binding to BRD3(2) H395R, the studied mutations are still inhibited by two diverse BET inhibitors and it is therefore not expected that the studied mutants will lead to drug resistance.

The results obtained from the study on BRDs nsSNPS may raise additional questions since BRDs are involved in the activation of oncogenes expression and most of the variants are less stable than the wild type, thus the variants might be expected to be less “deleterious”. Considering the pivotal role of BET proteins in the regulation of the transcription of growth-promoting genes and cell cycle regulators, the phenotypic perturbations of BRDs variants may potentially lead to oncogene activation thus significantly affect the tumour development [4].

## Materials and Methods

### Plasmids and site-directed mutagenesis

The plasmids harboring the BRDs wild type genes [14] were used to obtain mutant enzymes. The wild type plasmids were subjected to site-directed mutagenesis using Quick Change Site-directed Mutagenesis Kit (Stratagene), combined with specific sense and antisense mutagenic oligonucleotides as shown in S4 Table. The presence of the desired mutations and the absence of unwanted additional mutations were confirmed by inserts sequencing.

### Protein expression and purification

Wild type and mutant proteins were expressed in *E*.*coli*. Rosetta cells transformed with the selected plasmids were grown at 37°C in LB medium with Kan antibiotic to an OD_595_ = 0.6. Upon reached desired OD temperature was lowered up to 18°C, cultures were grown overnight after induction with 0.5 mM isopropyl-β-D-thiogalactopyranoside. Protein purification was carried out at 4°C modifying the protocol indicated by Filippakopoulos *et al*. [11]. Cells from one liter culture were collected by centrifugation, re-suspended in 50 ml of buffer A (50 mM HEPES, pH 7.5, 0.5 M NaCl, 5% Glycerol and 0.5 mM tris(2-carboxyethyl)phosphine–HCl (TCEP) containing a cocktail of EDTA-free protease inhibitors (Roche), sonicated in a Vibracell 75115 sonicator with 5 s boosts and 9 s pause, on ice. The sonicated cells were centrifuged and the supernatant, after an additional centrifugation at 15000 rpm, was applied to a 5 ml prepacked His Trap column (GE Healthcare) equilibrated in buffer A. The His- tagged fusion protein was eluted with 0.25 M imidazole in buffer A. The eluted protein was concentrated to a final volume of 2.5 ml on an Amicon concentrator Ultra-15 (Millipore) and then applied to a PD-10 pre-packed column (GE Healthcare) to remove imidazole. The protein in buffer A was cleaved by recombinant His-tagged tobacco etch virus (TEV) protease (kindly provided by SK), overnight at 4°C. The digested mixture containing TEV protease, the His-tag and the cleaved protein was applied to a 5 ml pre-packed His Trap column (GE Healthcare) previously equilibrated in buffer A. The flow through containing the protein without His-tag was collected, and checked for purity and size by SDS–PAGE on a pre-casted NuPage 4–12% bis-Tris polyacrylamide gel (Invitrogen) (S7 and S8 Figs). Gels were stained with Coomassie blue R-250. The protein without His-tag was used for all structural and stability experiments. The mutant and wild type enzymes obtained were approximately 70 mg from one liter culture. Protein quantification was determined according to OD_280_ measurement using respective molar extinction coefficients ε of each protein, calculated according to Gill and Hippel [49].

### Spectroscopic measurements

For intrinsic fluorescence emission measurements, the absorbance of the protein solutions at 280 nm was 0.10 AU, corresponding to a protein concentration ranging over 50.0–100 μg/mL. Intrinsic fluorescence emission measurements were carried out at 10°C with a LS50B spectrofluorimeter (Perkin-Elmer) using a 1.0 cm path length quartz cuvette. Fluorescence emission spectra were recorded from 300 to 450 nm (1 nm sampling interval), with the excitation wavelength set at 295 nm. For far-UV (190–250 nm) CD spectra, the absorbance of the protein solutions at 280 nm was 0.18 AU, corresponding to a protein concentration ranging over 100–190 μg/ml (0.4 mM DTT, 0.1 cm path length quartz cuvette) or 0.10 AU, corresponding to a protein concentration ranging over 50.0–100 μg/ml. (0.2 mM DTT, 0.2 cm path length quartz cuvette). For near-UV (250–320 nm) CD spectra, the absorbance of the protein solutions at 280 nm was 2.2 AU, corresponding to a protein concentration ranging over 1.25–2.28 mg/mL (2.0 mM DTT, 1.0 cm path length quartz cuvette). The results obtained from CD measurements were expressed as the mean residue ellipticity ([Θ]), assuming a mean residue molecular mass of 110 per amino acid residue. All spectroscopic measurements were carried out at 10°C in 20 mM Tris-HCl pH 7.5 containing 0.20 M NaCl.

### Urea-induced equilibrium unfolding

For equilibrium transition studies, BRDs wild type and variants (final concentration ranging over 50.0–100 μg/ml) were incubated at 10°C at increasing concentrations of urea (0−8 M) in 20 mM Tris/HCl, pH 7.5, in the presence of 0.2 M NaCl and 200 μM DTT. After 10 min, equilibrium was reached and intrinsic fluorescence emission and far-UV CD spectra (0.2-cm cuvette) were recorded in parallel at 10°C. To test the reversibility of the unfolding, BRDs wild type and variants were unfolded at 10°C in 7.5 M urea at protein concentration ranging over 0.5–1.0 mg/ml in 20 mM Tris/HCl, pH 7.5, in the presence of 2 mM DTT and 0.2 M NaCl. After 10 min, refolding was started by 10-fold dilution of the unfolding mixture at 10°C into solutions of the same buffer used for unfolding containing decreasing urea concentrations. The final protein concentration ranged over 50.0–100 μg/mL. After 24 h, intrinsic fluorescence emission and far-UV CD spectra were recorded at 10°C. All denaturation experiments were performed in triplicate.

### Thermal denaturation experiments

BRDs variants and wild type (protein concentration ranging over 0.10–0.20 mg/ml) were heated from 20°C to 80°C in a 0.1 cm quartz cuvette with a heating rate of 1 degree x min^-1^ controlled by a Jasco programmable Peltier element. The dichroic activity at 222 nm and the PMTV were continuously monitored in parallel every 0.5°C [39]. All the thermal scans were corrected for the solvent contribution at the different temperatures. Melting temperature (Tm) values were calculated by taking the first derivative of the ellipticity at 222 nm with respect to temperature. All denaturation experiments were performed in triplicate.

### Bio-layer Interferometry

In Bio-layer Interferometry (BLI) experiment the affinity between histones acetylated peptides and the proteins was measured. The proteins (20 μM) were dialyzed against the assay buffer (25 mM Hepes pH 7.5, 100 mM NaCl and 0.01% TWEEN). The biotinylated acetylated peptides of the H2A, H2B, H3 and H4 histones were immobilized onto strepdavidin biosensor (FòrteBio). All binding experiments were conducted at 25°C using an OctetRed 384 instrument (FòrteBio). Common cycles steps for analysis included 120 s of biosensor baseline equilibration step, associations in wells containing the free label protein for 240 s, and dissociations in buffer wells for 240 s. Reference subtraction was performed with the FòrteBio data analysis software to subtract the effect of baseline drift and the effect of nonspecific binding to biosensor tips without immobilized peptides.

### Data analysis

Far-UV CD spectra recorded as a function of urea concentration were analyzed by a singular value decomposition algorithm (SVD) using the software MATLAB (Math-Works, South Natick, MA) to remove the high frequency noise and the low frequency random errors and determine the number of independent components in any given set of spectra. CD spectra in the 213–250 nm region were placed in a rectangular matrix *A* of *n* columns, one column for each spectrum collected at each time. The *A* matrix is decomposed by SVD into the product of three matrices: *A* = U*S*V^T^, where *U* and *V* are orthogonal matrices and *S* is a diagonal matrix. The *U* matrix columns contain the basis spectra and the *V* matrix columns contain the urea dependence of each basis spectrum. Both *U* and *V* columns are arranged in terms of decreasing order of the relative weight of information, as indicated by the magnitude of the singular values in *S*. The diagonal *S* matrix contains the singular values that quantify the relative importance of each vector in *U* and *V*. The signal-to-noise ratio is very high in the earliest columns of *U* and *V* while the random noise is mainly accumulated in the latest *U* and *V* columns. The wavelength averaged spectral changes induced by increasing denaturant concentrations are represented by the columns of matrix *V*; hence, the plot of the columns of *V* versus the denaturant concentrations provides information about the observed transition.

The changes in intrinsic fluorescence emission spectra at increasing urea concentrations were quantified as the intensity-averaged emission wavelength, $\bar{\lambda }$, [50] calculated according to

$$\bar{\lambda }=\sum \left(I_{\text{i}}\lambda_{\text{i}}\right)/\sum \left(I_{\text{i}}\right)$$ (1)

where λ_i_ and *I*_i_ are the emission wavelength and its corresponding fluorescence intensity at that wavelength, respectively. This quantity is an integral measurement, negligibly influenced by the noise, which reflects changes in the shape and position of the emission spectrum.

Urea-induced equilibrium unfolding transitions monitored by far-UV CD ellipticity and intrinsic fluorescence emission changes were analysed by fitting baseline and transition region data to a two-state linear extrapolation model [51] according to

$$\Delta G_{\text{unfolding}}=\Delta G^{{\text{H}}_{2}\text{O}}+m\left[\text{Urea}\right]=\;-RTlnK_{\text{unfolding}}$$ (2)

where Δ*G*_unfolding_ is the free energy change for unfolding for a given denaturant concentration, Δ*G*^H^_2_^O^ the free energy change for unfolding in the absence of denaturant and *m* a slope term which quantifies the change in Δ*G*_unfolding_ per unit concentration of denaturant, *R* the gas constant, *T* the temperature and *K*_unfolding_ the equilibrium constant for unfolding. The model expresses the signal as a function of denaturant concentration:

$$y_{i}=\frac{y_{\text{N}}+s_{\text{N}}{\left[X\right]}_{i}+\left(y_{\text{U}}+s_{\text{U}}{\left[X\right]}_{i}\right)*\text{exp}\left[\left(-\Delta G{\text{​}}^{H_{2}O}-m{\left[X\right]}_{i}\right)/RT\right]}{1+\text{exp}\left[\frac{\left(-\Delta G^{H_{2}O}-m{\left[X\right]}_{i}\right)}{RT}\right]}$$ (3)

where *y*_i_ is the observed signal, *y*_U_ and y_N_ are the baseline intercepts for unfolded and native protein, *s*_U_ and *s*_N_ are the baseline slopes for the unfolded and native protein, [X]_i_ the denaturant concentration after the ith addition, Δ*G*^H^_2_^O^ the extrapolated free energy of unfolding in the absence of denaturant, *m* the slope of a Δ*G*_unfolding_ versus [X] plot. The denaturant concentration at the midpoint of the transition, [Urea]_0.5_, according to Eq 2, is calculated as:

$$\;\;{\left[\text{Urea}\right]}_{0.5}=\Delta G^{{\text{H}}_{2}\text{O}}/m$$ (4)

The denaturation curve obtained by plotting the fluorescence changes of the BRD2(1) variant R100L induced by increasing urea concentrations was fitted to the following equation assuming a three-state model:

$$F=\frac{F_{\text{N}}+\text{exp}\left(m_{\text{I-N}}\frac{[\text{urea}]-D50_{\text{I-N}}}{RT}\right)•\left(F_{\text{I}}+F_{\text{U}}\text{exp}\left(m_{\text{U-I}}\frac{[\text{urea}]-D50_{\text{U-I}}}{RT}\right)\right)}{1+\text{exp}\left(m_{\text{I-N}}\frac{[\text{urea}]-D50_{\text{I-N}}}{RT}\right)•\left(1+\text{exp}\left(m_{\text{U-I}}\frac{[\text{urea}]-D50_{\text{U-I}}}{RT}\right)\right)}$$ (5)

where *F* is $\bar{\lambda }$, calculated according to Eq 1, *m* is a constant that is proportional to the increase in solvent-accessible surface area between the two states involved in the transition, *D*50_I-N_ and *m*_I-N_ are the midpoint and *m* value for the transition between N and I, respectively, and *D*50_U-I_ and *m*_U-I_ are the midpoint and *m* value for the transition between I and U, respectively [52]. The $\bar{\lambda }$ of the intermediate state (I), *F*_I_, is constant whereas that of the folded state (N) and of the unfolded state (U), *F*_N_ and *F*_U_, respectively, has a linear dependence on denaturant concentration

$$\;\;\;\;\;F_{\text{N}}=a_{\text{N}}+b_{\text{N}}\left[\text{urea}\right]$$ (6)

$$\;F_{\text{U}}=a_{\text{U}}+b_{\text{U}}\left[\text{urea}\right]$$ (7)

where *a*_N_ and *a*_U_ are the baseline intercepts for N and U, *b*_N_ and *b*_U_ are the baseline slopes for N and U, respectively. All unfolding transition data were fitted by using Graphpad Prism 5.04.

### Isothermal Titration Calorimetry

Isothermal titration calorimetry (ITC) experiments were performed at 20°C using a MicroCal VP-ITC calorimeter. BRDs proteins were extensively dialyzed with an Amicon Ultrafiltration device against the assay buffer (50 mM Hepes, pH 7.5, 100 mM NaCl, 0.5 mM TCEP). The assay buffer was also used to dilute the inhibitors 50 mM DMSO stock solutions to the experiment final concentration (15 μM). In all the experiments the inhibitors JQ1 or PFI-1 were placed in the sample cell under continuous stirring while the protein solution (ranging concentration between 200 and 250 μM) was loaded into the syringe injector. The titrations curves implied 30 injections of 6 μL at 180 s intervals. The thermodynamic data were processed with Origin 7.0 software provided by MicroCal.

### Protein crystallization and structure determination

Aliquots of the purified proteins were set up for crystallization using a mosquito^®^ crystallization robot (TTP Labtech). Coarse screens were typically setup onto Greiner 3-well plates using three different drop ratios of precipitant to protein per condition (100+50 nL, 75+75 nL and 50+100 nL). All crystallizations were carried out using the sitting drop vapour diffusion method at 4°C and were crystallized as described [14]. Crystals were cryo-protected using the well solution supplemented with additional ethylene glycol and were flash frozen in liquid nitrogen. Data were collected at diamond beamline I04 at a wavelength of 1.0121 Å. Indexing and integration was carried out using MOSFLM [53] and scaling was performed with SCALA [54]. Initial phases were calculated by molecular replacement with PHASER [55] using available structures of wild type proteins [14]. Initial models were built by ARP/wARP [56] and building was completed manually with COOT [57]. Refinement was carried out in REFMAC5 [58]. Thermal motions were analyzed using TLSMD [59] and hydrogen atoms were included in late refinement cycles.

## Supporting Information

S1 Fig Intrinsic fluorescence emission spectra of wild type bromodomains and mutants. Intrinsic fluorescence emission spectra were recorded at 20°C in 20 mM Tris/HCl, pH 7.5 containing 0.20 M NaCl and 200 μM DTT (295 nm excitation excitation wavelength) as described in Materials and Methods. The absorbance at 280 nm was 0.10 AU for all the protein solutions. Wild type spectra are shown as black solid lines and mutants are coloured as indicated in the figure.
(TIF)

S2 Fig Thermal unfolding transition of BRD2(1) and BRD4(1) wild type and variants studied by CD spectroscopy. Wild type and variants were heated from 20°C to 80°C in a 0.1-cm quartz cuvette at 0.2 mg/ml in 20 mM Tris/HCl, pH 7.5 containing 0.20 M NaCl and 0.40 mM DTT and the molar ellipticity at 222 nm ([Θ_222_]) was monitored continuously every 0.5°C. Normalized [Θ_222_] of BRD2(1) (A), BRD4 (1) (B); the insets show the first derivative of the same data as in (A) and in (B). (C) and (D) [Θ_222_] before normalization.
(TIF)

S3 Fig Thermal unfolding transition of BRD2(2), BRD3(2) and BRD4(2) wild type and variants studied by CD spectroscopy. Wild type and variants were heated from 20°C to 80°C in a 0.1-cm quartz cuvette at 0.2 mg/ml in 20 mM Tris/HCl, pH 7.5 containing 0.20 M NaCl and 0.40 mM DTT and the molar ellipticity at 222 nm ([Θ_222_]) was monitored continuously every 0.5°C. Normalized [Θ_222_] of BRD2(2) (A), BRD3 (2) (B), BRD4(2) (C); the insets show the first derivative of the same data as in (A), (B) and in (C). (D), (E) and (F) [Θ_222_] before normalization.
(TIF)

S4 FigUrea-induced equilibrium unfolding of BRD2(1) and BRD4(1) wild type and variants.(A) and (B) Normalized molar ellipticity at 222 nm ([Θ]_222_) reported after removal of the high-frequency noise and the low-frequency random error by SVD; (C) and (D) Normalized intensity-averaged emission wavelength ($\bar{\lambda }$). The continuous lines represent the nonlinear fitting of the normalized molar ellipticities at 222 nm and of the normalized intensity-averaged emission wavelength data to Eq 3, at increasing denaturant concentrations, calculated as described in Materials and Methods. The inset in (C) shows the three-state unfolding of BRD2(1) R100L variant fitted according to Eq 5. The reversibility points (empty circles) are shown, for clarity, only for the wild type and for R100L and were not included in the nonlinear regression analysis. All the spectra were recorded at 10°C as described in Materials and Methods.(TIF)

S5 FigUrea-induced equilibrium unfolding of BRD2(2), BRD3(2) and BRD4(2) wild type and variants.(A), (B) and (C) Normalized molar ellipticity at 222 nm ([Θ]_222_) reported after removal of the high-frequency noise and the low-frequency random error by SVD; (D), (E) and (F) normalized intensity-averaged emission wavelength ($\bar{\lambda }$). The continuous lines represent the nonlinear fitting of the normalized molar ellipticities at 222 nm and of the normalized intensity-averaged emission wavelength data to Eq 3, at increasing denaturant concentrations, calculated as described in Materials and Methods. The reversibility points (empty circles) are shown, for clarity, only for the wild type and were not included in the nonlinear regression analysis. All the spectra were recorded at 10°C as described in Materials and Methods.(TIF)

S6 Fig Details of the environment of residues variants in BRD2. Shown is a structural overview (top left) and details of interactions with neighbouring residues within a radius of 6 Å. The mutated residues are shown in ball and stick representation and main structural elements are labeled.
(PDF)

S7 Fig SDS-PAGE analysis of BRD2(1) and BRD4(1) wild type and variants. Lane 1, protein molecular mass markers; lane 2, wild type protein with His-tag; lane 3, wild type protein after overnight treatment with TEV protease; lane 4, purified wild type protein without His-tag. (A) BRD2(1) lane 5–11, purified variants without His-tag (15 kDa); (B) BRD4(1) lane 5, purified variant without His-tag (15 kDa). All the proteins were cleaved by TEV protease overnight at 4°C and purified on a His Trap column. The flow through containing the purified proteins without His-tag was collected and analyzed by SDS–PAGE. Gels were stained with Coomassie blue R-250.
(PDF)

S8 Fig SDS-PAGE analysis of BRD2(2), BRD3(2) and BRD4(2) wild type and variants. Lane 1, protein molecular mass markers; lane 2, wild type protein with His-tag; lane 3, wild type protein after overnight treatment with TEV protease; lane 4, purified wild type protein without His-tag. (A) BRD2(2) lane 5 and 6, purified variants without His-tag (13 kDa); (B) BRD3(2) lane 5, purified variant without His-tag (13 kDa); (C) BRD4(2) lane 5, purified variant without His-tag (15 kDa). All the proteins were cleaved by TEV protease overnight at 4°C and purified on a His Trap column. The flow through containing the purified proteins without His-tag was collected and analyzed by SDS–PAGE. Gels were stained with Coomassie blue R-250.
(PDF)

S1 Table Data collection and refinement statistics. (PDF)

S2 Table Dissociation constants and thermodynamic parameters from isothermal titration calorimetry assays. In all cases proteins were titrated into the ligand solution (reverse titration).
(PDF)

S3 Table Structural location and difference in melting temperature and in free energy of urea-induced unfolding of BRDs wild type and mutants. (PDF)

S4 Table List of oligonucleotides used for site-directed mutagenesis. (PDF)

## Abbreviations

Kac: acetylated Lysine residues
BRD: Bromodomain
BET: Bromo and Extra Terminal
nsSNP: nonsynonymous single nucleotide polymorphism
BLI: biolayer interference
ITC: isothermal titration calorimetry

## References

[1] Holliday R. The inheritance of epigenetic defects. Science. 1987; 238: 163–170 3310230 10.1126/science.3310230

[2] Dawson MA, Kouzarides T, Huntly BJ. Targeting epigenetic readers in cancer. New England Journal of Medicine. 2012; 367: 647–657 10.1056/NEJMra1112635 22894577

[3] Filippakopoulos P, Knapp S. Targeting bromodomains: epigenetic readers of lysine acetylation. Nature Reviews Drug Discovery. 2014; 13: 337–356 10.1038/nrd4286 24751816

[4] Muller S, Filippakopoulos P, Knapp S. Bromodomains as therapeutic targets. Expert Reviews in Molecular Medicine. 2011; 13 e29 10.1017/S1462399411001992 21933453 PMC3177561

[5] Dawson MA, Kouzarides T. Cancer epigenetics: from mechanism to therapy. Cell. 2012;150: 12–27 10.1016/j.cell.2012.06.013 22770212

[6] Hewings DS, Rooney TP, Jennings LE, Hay DA, Schofield CJ, Brennan PE, et al Progress in the development and application of small molecule inhibitors of bromodomain-acetyl-lysine interactions. Journal of Medicinal Chemistry. 2012; 55:9393–9413. 10.1021/jm300915b 22924434

[7] Kumar R, Li DQ, Müller, Knapp S. Epigenomic regulation of oncogenesis by chromatin remodeling. Oncogene. 2016 1 25 10.1038/onc.2015.513 [Epub ahead of print] 26804164

[8] Martin LJ, Koegl M, Bader G, Cockcroft XL, Fedorov O, Fiegen D et al Structure-based design of an in vivo active selective BRD9 inhibitor. Journal of Medicinal Chemistry. 2016; 59: 4462–4475. 10.1021/acs.jmedchem.5b01865 26914985 PMC4885110

[9] Sutherell CL, Tallant C, Monteiro OP, Yapp C, Fuchs JE, Fedorov O, et al Identification and development of 2,3-Dihydropyrrolo[1,2-a]quinazolin-5(1H)-one inhibitors targeting bromodomains within the switch/sucrose nonfermenting complex. Journal of Medicinal Chemistry. 2016; 59: 5095–5101. 10.1021/acs.jmedchem.5b01997 27119626 PMC4920105

[10] Delmore JE, Issa GC, Lemieux ME, Rahl PB, Shi J, Jacobs HM, et al BET Bromodomain Inhibition as a Therapeutic Strategy to Target c-Myc. Cell. 2011; 146: 904–917 10.1016/j.cell.2011.08.017 21889194 PMC3187920

[11] Filippakopoulos P, Qi J, Picaud S, Shen Y, Smith WB, Fedorov O, et al Selective inhibition of BET bromodomains. Nature. 2010; 468: 1067–1073 10.1038/nature09504 20871596 PMC3010259

[12] Zuber J, Shi J, Wang E, Rappaport AR, Herrmann H, Sison EA, et al RNAi screen identifies Brd4 as a therapeutic target in acute myeloid leukaemia. Nature. 2011; 478: 524–528 10.1038/nature10334 21814200 PMC3328300

[13] Jung M, Gelato KA, Fernandez-Montalvan A, Siegel S, Haendler B. Targeting BET bromodomains for cancer treatment. Epigenomics. 2015; 7: 487–501 10.2217/epi.14.91 26077433

[14] Filippakopoulos P, Picaud S, Mangos M, Keates T, Lambert JP, Barsyte-Lovejoy D, et al Histone recognition and large-scale structural analysis of the human bromodomain family. Cell. 2012; 149: 214–231 10.1016/j.cell.2012.02.013 22464331 PMC3326523

[15] Owen DJ, Ornaghi P, Yang JC, Lowe N, Evans PR, Ballario P, et al The structural basis for the recognition of acetylated histone H4 by the bromodomain of histone acetyltransferase gcn5p. The Embo Journal. 2000; 19: 6141–6149 11080160 10.1093/emboj/19.22.6141 PMC305837

[16] Dey A, Chitsaz F, Abbasi A, Misteli T, Ozato K. The double bromodomain protein Brd4 binds to acetylated chromatin during interphase and mitosis. Proceedings of the National Academy of Sciences of the United States of America. 2003; 100: 8758–8763 12840145 10.1073/pnas.1433065100 PMC166386

[17] Sinha A, Faller DV, Denis GV. Bromodomain analysis of Brd2-dependent transcriptional activation of cyclin A. Biochemical Journal. 2005; 387: 257–269 15548137 10.1042/BJ20041793 PMC1134954

[18] LeRoy G, Rickards B, Flint SJ. The double bromodomain proteins Brd2 and Brd3 couple histone acetylation to transcription. Molecular Biology of the Cell. 2008; 30: 51–60 10.1016/j.molcel.2008.01.018 PMC2387119 18406326

[19] Nishiyama A, Dey A, Miyazaki J, Ozato K. Brd4 is required for recovery from antimicrotubule drug-induced mitotic arrest: preservation of acetylated chromatin. Molecular Biology of the Cell. 2006; 17: 814–823 16339075 10.1091/mbc.E05-08-0729 PMC1356591

[20] Yang Z, He N, Zhou Q. Brd4 recruits P-TEFb to chromosomes at late mitosis to promote G1 gene expression and cell cycle progression. Molecular Biology of the Cell. 2008; 28: 967–976 10.1128/MCB.01020-07 PMC2223388 18039861

[21] Belkina AC, Blanton WP, Nikolajczyk BS, Denis GV. The double bromodomain protein Brd2 promotes B cell expansion and mitogenesis. Journal of Leukocyte Biology. 2014; 95: 451–460 10.1189/jlb.1112588 24319289 PMC3923082

[22] French CA. Pathogenesis of NUT midline carcinoma. Annual Review of Pathology. 2012; 7: 247–265 10.1146/annurev-pathol-011811-132438 22017582

[23] French CA, Kutok JL, Faquin WC, Toretsky JA, Antonescu CR, Griffin CA, et al Midline carcinoma of children and young adults with NUT rearrangement. Journal of Clinical Oncology. 2004; 22: 4135–4139 15483023 10.1200/JCO.2004.02.107

[24] French CA, Miyoshi I, Aster JC, Kubonishi I, Kroll TG, Dal C, et al BRD4 bromodomain gene rearrangement in aggressive carcinoma with translocation t(15;19). American Journal of Pathology. 2001; 159: 1987–1992 11733348 10.1016/S0002-9440(10)63049-0 PMC1850578

[25] Ishii H, Mimori K, Mori M, Vecchione A. Differentially expressed genes in endothelial differentiation. DNA and Cell Biology. 2005; 24: 432–437 16008511 10.1089/dna.2005.24.432

[26] Zhou M, Peng C, Nie XM, Zhang BC, Zhu SG, Yu Y, et al Expression of BRD7-interacting proteins BRD2 and BRD3 in nasopharyngeal carcinoma tissues. Ai Zheng 2003; 22: 123–127 12600283

[27] Lori C, Lantella A, Pasquo A, Alexander LT, Knapp S, Chiaraluce R, et al Effect of single amino acid substitution observed in cancer on Pim-1 kinase thermodynamic stability and structure. PLoS One 2013; 8 e64824 10.1371/journal.pone.0064824 23755147 PMC3673989

[28] Pasquo A, Consalvi V, Knapp S, Alfano I, Ardini M, Stefanini S, et al Structural stability of human protein tyrosine phosphatase rho catalytic domain: effect of point mutations PLoS One 2012;7 e32555 10.1371/journal.pone.0032555 22389709 PMC3289658

[29] Casadio R, Vassura M, Tiwari S, Fariselli P, Luigi Martelli P. Correlating disease-related mutations to their effect on protein stability: a large-scale analysis of the human proteome. Human Mutation. 2011; 32: 1161–1170 10.1002/humu.21555 21853506

[30] Kucukkal TG, Petukh M, Li L, Alexov E. Structural and physico-chemical effects of disease and non-disease nsSNPs on proteins. Current Opinion in Structural Biology. 2015; 32: 18–24 10.1016/j.sbi.2015.01.003 25658850 PMC4511717

[31] Fong CY, Gilan O, Lam EY, Rubin AF, Ftouni S, Tyler D, et al BET inhibitor resistance emerges from leukaemia stem cells. Nature. 2015; 525: 538–542 10.1038/nature14888 26367796 PMC6069604

[32] Rathert P, Roth M, Neumann T, Muerdter F, Roe JS, Muhar M, et al Transcriptional plasticity promotes primary and acquired resistance to BET inhibition. Nature. 2015; 525: 543–547 10.1038/nature14898 26367798 PMC4921058

[33] Forbes SA, Bindal N, Bamford S, Cole C, Kok CY, Beare D et al COSMIC: mining complete cancer genomes in the Catalogue of Somatic Mutations in Cancer. Nucleic Acids Research 2011; 39 D945–950 10.1093/nar/gkq929 20952405 PMC3013785

[34] Choy N, Raussens V, Narayanaswami V. Inter-molecular coiled-coil formation in human apolipoprotein E C-terminal domain. Jornal of Molecular Biology. 2003;334: 527–539 10.1016/j.jmb.2003.09.059 14623192

[35] Kiss RS, Weers PM, Narayanaswami V, Cohen J, Kay CM, Ryan RO. Structure-guided protein engineering modulates helix bundle exchangeable apolipoprotein properties. Journal of Biological Chemistry. 2003; 278: 21952–21959 12684504 10.1074/jbc.M302676200

[36] Lamonica JM, Deng W, Kadauke S, Campbell AE, Gamsjaeger R, Wang H, et al Bromodomain protein Brd3 associates with acetylated GATA1 to promote its chromatin occupancy at erythroid target genes. Proceedings of the National Academy of Sciences of the United States of America. 2011; 108: E159–168 10.1073/pnas.1102140108 21536911 PMC3107332

[37] Fish PV, Filippakopoulos P, Bish G, Brennan PE, Bunnage ME, Cook AS, et al Identification of a chemical probe for bromo and extra C-terminal bromodomain inhibition through optimization of a fragment-derived hit. Journal of Medicinal Chemistry. 2012; 55: 9831–9837 10.1021/jm3010515 23095041 PMC3506127

[38] Picaud S, Da Costa D, Thanasopoulou A, Filippakopoulos P, Fish PV, Philpott M, et al PFI-1 a highly selective protein interaction inhibitor targeting BET Bromodomains. Cancer Research. 2013;73: 3336–3346 10.1158/0008-5472.CAN-12-3292 23576556 PMC3673830

[39] Benjwal S, Verma S, Rohm KH, Gursky O. Monitoring protein aggregation during thermal unfolding in circular dichroism experiments. Protein Science. 2006;15: 635–639 16452626 10.1110/ps.051917406 PMC2249783

[40] Pradeep L, Udgaonkar JB. Effect of salt on the urea-unfolded form of barstar probed by m value measurements. Biochemistry. 2004;43: 11393–11402 15350126 10.1021/bi049320b

[41] Shortle D. Staphylococcal nuclease: a showcase of m-value effects. Advances in Protein Chemistry. 1995; 46: 217–247 7771319 10.1016/s0065-3233(08)60336-8

[42] Wrabl J, Shortle D. A model of the changes in denatured state structure underlying m value effects in staphylococcal nuclease. Nature Structural Biology. 1999;6: 876–883 10467101 10.1038/12338

[43] Spudich G, Marqusee S. A change in the apparent m value reveals a populated intermediate under equilibrium conditions in Escherichia coli ribonuclease HI. Biochemistry. 2000;39: 11677–11683 10995235 10.1021/bi000466u

[44] Geierhaas CD, Nickson AA, Lindorff-Larsen K, Clarke J, Vendruscolo M. BPPred: a Web-based computational tool for predicting biophysical parameters of proteins Protein Science. 2007; 16: 125–134 17123959 10.1110/ps.062383807 PMC2222837

[45] Fuchs JE, Waldner BJ, Huber RG, von Grafenstein S, Kramer C, Liedl KR. Independent Metrics for Protein Backbone and Side-Chain Flexibility: Time Scales and Effects of Ligand Binding. Journal of Chemical Theory and Computation. 2015; 11: 851–860. 10.1021/ct500633u 26579739

[46] Gaboriau DC, Rowling PJ, Morrison CG, Itzhaki LS. Protein stability versus function: effects of destabilizing missense mutations on BRCA1 DNA repair activity. Biochemical Journal. 2015; 466: 613–624 10.1042/BJ20141077 25748678

[47] Gao M, Zhou H, Skolnick J. Insights into disease-associated mutations in the human proteome through protein structural analysis. Structure. 2015; 23: 1362–1369 10.1016/j.str.2015.03.028 26027735 PMC4497952

[48] Engin HB, Kreisberg JF, Carter H. Structure-based analysis reveals cancer missense mutations target protein interaction interfaces.PLoS One. 2016 4 4;11(4). 10.1371/journal.pone.0152929 PMC4820104 27043210

[49] Gill SC, von Hippel PH. Calculation of protein extinction coefficients from amino acid sequence data. Analytical Biochemistry. 1989; 182: 319–326 2610349 10.1016/0003-2697(89)90602-7

[50] Royer CA, Mann CJ, Matthews CR. Resolution of the fluorescence equilibrium unfolding profile of trp aporepressor using single tryptophan mutants. Protein Science. 1993; 2: 1844–1852 8268795 10.1002/pro.5560021106 PMC2142281

[51] Santoro MM, Bolen DW. Unfolding free energy changes determined by the linear extrapolation method. 1. Unfolding of phenylmethanesulfonyl alpha-chymotrypsin using different denaturants. Biochemistry 1988; 27: 8063–8068 3233195 10.1021/bi00421a014

[52] Rowling PJ, Cook R, Itzhaki LS. Toward classification of BRCA1 missense variants using a biophysical approach. Journal of Biological Chemistry 2010; 285: 20080–20087 10.1074/jbc.M109.088922 20378548 PMC2888420

[53] Leslie AGW, Powell H. 2007; MOSFLM (Cambridge: MRC Laboratory of Molecular Biology)

[54] Evans P 2007; SCALA—scale together multiple observations of reflections (Cambridge: MRC Laboratory of Molecular Biology)

[55] McCoy AJ, Grosse-Kunstleve RW, Storoni LC, Read RJ. Likelihood-enhanced fast translation functions. Acta Crystallographica Section D Biological Crystallography. 2005; 61: 458–464 15805601 10.1107/S0907444905001617

[56] Perrakis A, Morris R, Lamzin VS. Automated protein model building combined with iterative structure refinement. Nature Structural Biology. 1999; 6: 458–463 10331874 10.1038/8263

[57] Emsley P, Cowtan K. Coot: model-building tools for molecular graphics. Acta Crystallographica Section D Biological Crystallography 2004; 60: 2126–2132 15572765 10.1107/S0907444904019158

[58] Murshudov GN, Vagin AA, Dodson EJ. Refinement of macromolecular structures by the maximum-likelihood method. Acta Crystallographica Section D Biological Crystallography. 1997; 53: 240–255 15299926 10.1107/S0907444996012255

[59] Painter J, Merritt EA. Optimal description of a protein structure in terms of multiple groups undergoing TLS motion. Acta Crystallographica Section D Biological Crystallography. 2006; 62: 439–450 16552146 10.1107/S0907444906005270

