# Supplementary figures and images for: Effect of BET Missense Mutations on Bromodomain Function, Inhibitor Binding and Stability
Source: PLoS One. 2016 Jul 12;11(7):e0159180. doi: 10.1371/journal.pone.0159180 (PMC4942050; doi:10.1371/journal.pone.0159180)

**S6 Fig**

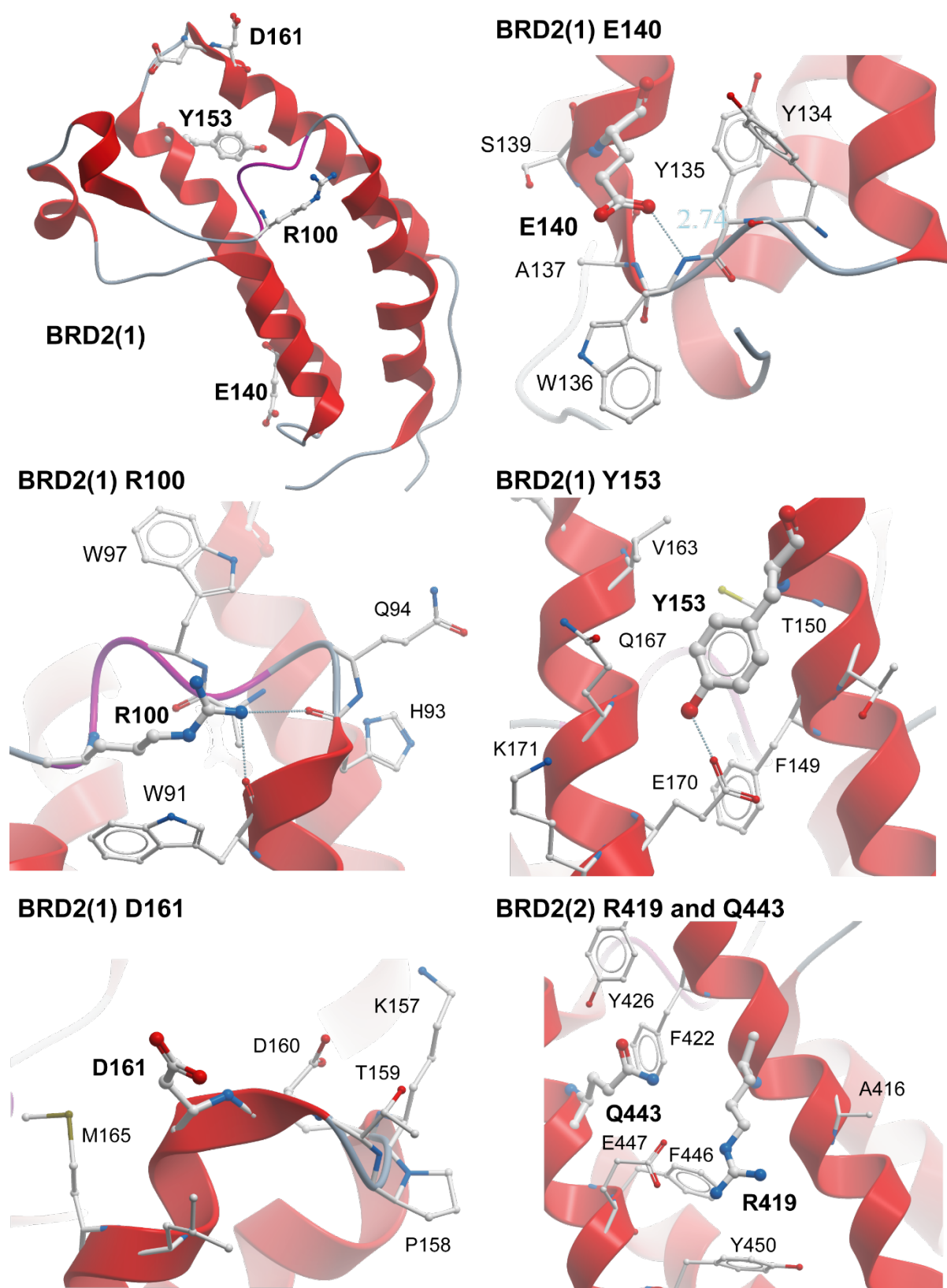

Supplement: S6 Fig — Shown is a structural overview (top left) and details of interactions with neighbouring residues within a radius of 6 Å. The mutated residues are shown in ball and stick representation and main structural elements are labeled. (PDF) [file pone.0159180.s006.pdf]

## (A) BRD2(1)

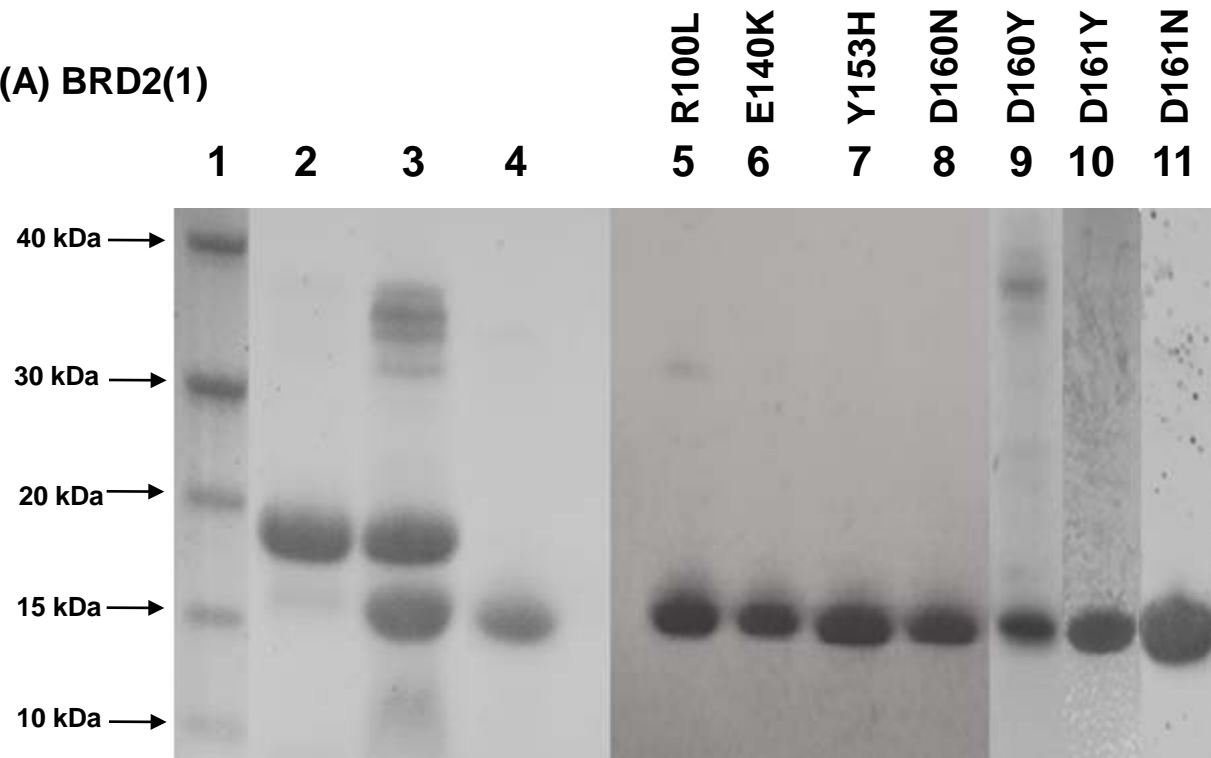

## (B) BRD4(1)

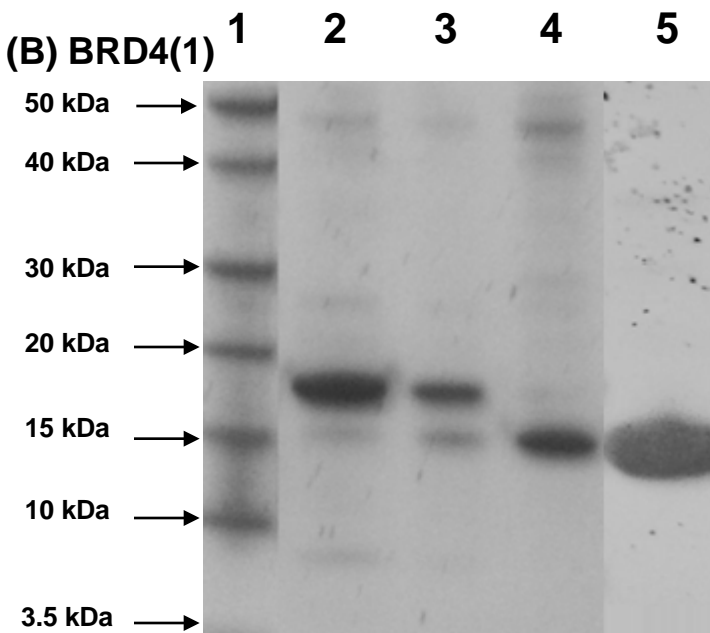

Supplement: S7 Fig — Lane 1, protein molecular mass markers; lane 2, wild type protein with His-tag; lane 3, wild type protein after overnight treatment with TEV protease; lane 4, purified wild type protein without His-tag. (A) BRD2(1) lane 5–11, purified variants without His-tag (15 kDa); (B) BRD4(1) lane 5, purified variant without His-tag (15 kDa). All the proteins were cleaved by TEV protease overnight at 4°C and purified on a His Trap column. The flow through containing the purified proteins without His-tag was collected and analyzed by SDS–PAGE. Gels were stained with Coomassie blue R-250. (PDF) [file pone.0159180.s007.pdf]

# S8 Fig

(A) BRD2(2)

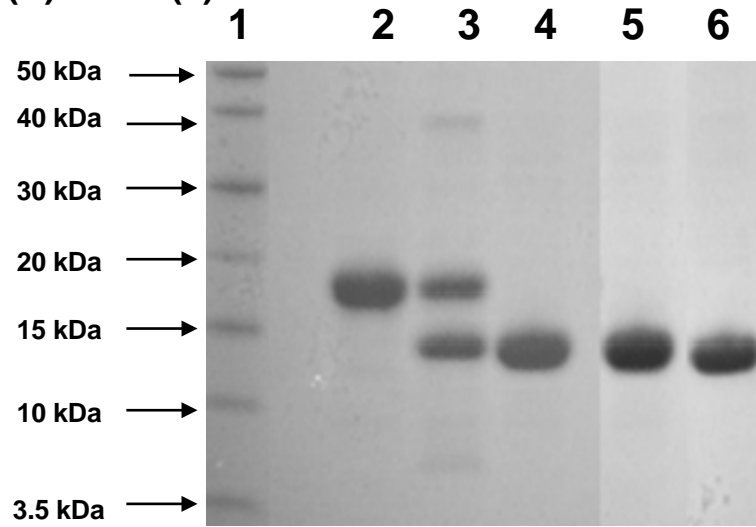

(B) BRD3(2)

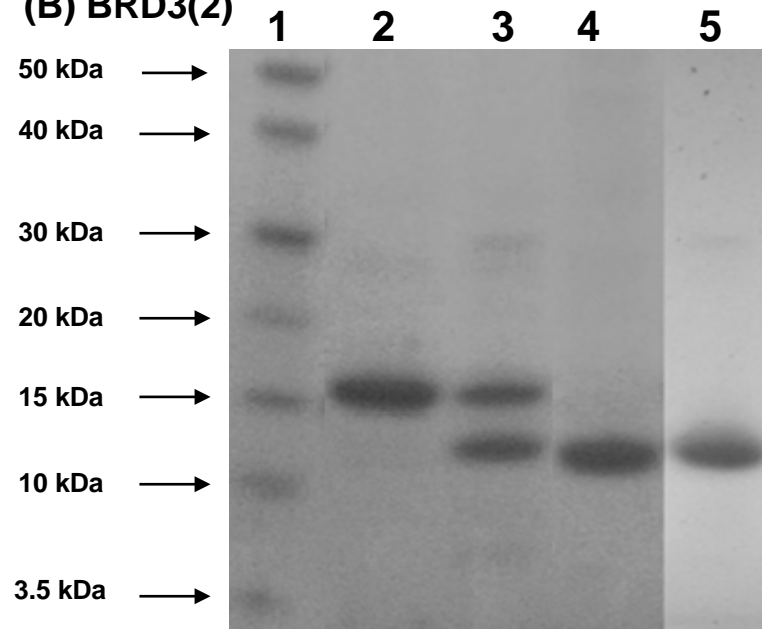

(C) BRD4(2)

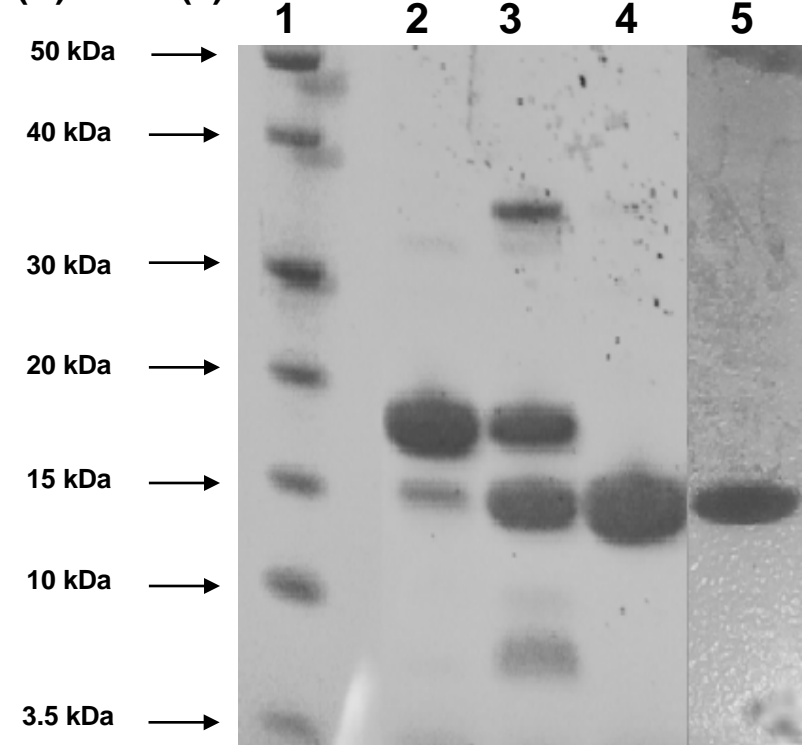

Supplement: S8 Fig — Lane 1, protein molecular mass markers; lane 2, wild type protein with His-tag; lane 3, wild type protein after overnight treatment with TEV protease; lane 4, purified wild type protein without His-tag. (A) BRD2(2) lane 5 and 6, purified variants without His-tag (13 kDa); (B) BRD3(2) lane 5, purified variant without His-tag (13 kDa); (C) BRD4(2) lane 5, purified variant without His-tag (15 kDa). All the proteins were cleaved by TEV protease overnight at 4°C and purified on a His Trap column. The flow through containing the purified proteins without His-tag was collected and analyzed by SDS–PAGE. Gels were stained with Coomassie blue R-250. (PDF) [file pone.0159180.s008.pdf]
