# Supplementary material for: Effect of BET Missense Mutations on Bromodomain Function, Inhibitor Binding and Stability
Source: PLoS One. 2016 Jul 12;11(7):e0159180. doi: 10.1371/journal.pone.0159180 (PMC4942050; doi:10.1371/journal.pone.0159180)
Supplement: S1 Table — (PDF) [file pone.0159180.s009.pdf]

S1Table. Data collection and refinement statistics

PDB ID	5HEL	5HEM	5HEN	5HFQ	5HFR
Protein / mutant	BRD2(1) / Y153H	BRD2(1) / D161Y	BRD2(1) / R100L	BRD2(2) / Q443H	BRD3(2) / H395R
Space group	$P\ 4_3\ 2_1\ 2$	$P\ 4_3\ 2_1\ 2$	C 1 2 1	$P\ 2\ 2_1\ 2_1$	$P\ 2_1\ 2\ 2_1$
Cell dimensions:					
a, b, c (Å)	47.74 47.74 125.79	68.73 68.73 113.36	115.38 55.76 68.45	32.02 52.81 72.31	64.83 92.55 102.95
α, β, γ (deg)	90.00 90.00 90.00	90.00 90.00 90.00	90.00 94.76 90.00	90.00 90.00 90.00	90.00 90.00 90.00
Resolution* (Å)	29.75 (1.45)	29.67 (1.65)	29.44 (1.79)	29.28 (1.40)	29.55 (1.70)
Unique observations*	26847 (3793)	33564 (4733)	40528 (2236)	24828 (1130)	68935 (3165)
Completeness* (%)	99.9 (99.2)	99.7 (99.4)	99.5 (93.5)	99.7 (95.8)	99.3 (87.4)
Redundancy*	13.7 (12.9)	6.0 (4.3)	5.9 (5.5)	12.3 (7.8)	12.7 (10.3)
Rmerge*	0.044 (0.336)	0.097 (0.373)	0.063 (0.790)	0.040 (0.063)	0.234 (2.483)
$I/\sigma I$*	34.2 (9.8)	13.8 (3.8)	14.2 (2.0)	48.5 (22.8)	13.0 (2.7)
**Refinement**					
Resolution (Å)	1.45	1.65	1.79	1.40	1.70
R_{work} / R_{free} (%)	19.09 / 20.90	16.67 (19.89)	18.08 (22.27)	16.68 (18.53)	19.37 (22.09)
Number of atoms					
(protein/other/water)	989 / 12 / 10	1982 / 29 / 214	2713 / 12 / 148	920 / 0 / 169	3634 / 20 / 402
B-factors (A^2)					
(protein/other/water)	18.04/26.93/29.27	15.52/22.23/24.35	35.84/39.32/38.13	9.68/0/22.08	19.38/32.81/28.94
r.m.s.d bonds (Å)	0.012	0.017	0.021	0.009	0.013
r.m.s.d angles ($^{o)}$	1.479	1.664	1.902	1.306	1.486
Ramachandran					
Favoured (%)	100.00	100.00	99.38	100.00	98.61
Allowed (%)	0.00	0.00	0.62	0.00	1.39
Disallowed (%)	0.00	0.00	0.00	0.00	0.00

* Values in parentheses correspond to the highest resolution shell
