# Supplementary material for: Effect of BET Missense Mutations on Bromodomain Function, Inhibitor Binding and Stability
Source: PLoS One. 2016 Jul 12;11(7):e0159180. doi: 10.1371/journal.pone.0159180 (PMC4942050; doi:10.1371/journal.pone.0159180)
Supplement: S2 Table — In all cases proteins were titrated into the ligand solution (reverse titration). (PDF) [file pone.0159180.s010.pdf]

**S2 Table**. Dissociation constants and thermodynamic parameters from isothermal titration calorimetry assays. In all cases proteins were titrated into the ligand solution (reverse titration).

| Comp | Protein | Mutant | $K_A$ ($10^6$ $M^{-1}$) | $K_D$ (nM) | N | Δ$H$ (kcal/mol) | TΔ$S$ (kcal/mol) * | Δ$G$ (kcal/mol) |
|---|---|---|---|---|---|---|---|---|
| JQ1 | BRD2(1) | wt | 20.9 ± 0.7 | 47.8 ± 1.6 | 0.875 ± 0.001 | -8.826 ± 0.017 | 0.976 | -9.802 |
| | BRD2(1) | D161N | 14.3 ± 0.8 | 69.9 ± 3.9 | 0.978 ± 0.002 | -6.877 ± 0.025 | 2.673 | -9.550 |
| | BRD2(1) | D161Y | 57.8 ± 3.0 | 17.3 ± 0.9 | 1.100 ± 0.001 | -7.356 ± 0.016 | 2.997 | -10.353 |
| | BRD2(1) | Y153H | 18.0 ± 0.8 | 55.5 ± 2.6 | 0.899 ± 0.002 | -9.689 ± 0.031 | 0.044 | -20.09 |
| | BRD2(1) | E140K | 26.0 ± 0.9 | 38.5 ± 1.4 | 0.933 ± 0.009 | -7.574 ± 0.015 | 2.330 | -9.904 |
| | BRD2(1) | R100L | 35.8 ± 1.7 | 27.9 ± 1.3 | 1.090 ± 0.001 | -7.918 ± 0.017 | 2.175 | -10.093 |
| | BRD2(2) | wt | 19.7 ± 1.4 | 50.8 ± 3.7 | 0.908 ± 0.002 | -4.368 ± 0.019 | 5.331 | -9.699 |
| | BRD2(2) | Q443H | 42.4 ± 3.8 | 23.6 ± 2.1 | 0.913 ± 0.002 | -5.169 ± 0.002 | 4.985 | -10.154 |
| | BRD2(2) | R419W | 33.4 ± 2.4 | 29.9 ± 2.2 | 1.100 ± 0.0019 | -3.274 ± 0.011 | 6.714 | -9.988 |
| | BRD3(2) | wt | 42.7 ± 2.4 | 23.4 ± 1.3 | 0.902 ± 0.001 | -6.930 ± 0.017 | 3.256 | -10.186 |
| | BRD3(2) | H395R | 142.8 ± 8.2 | 7.0 ± 0.4 | 1.100 ± 0.001 | -6.606 ± 0.011 | 4.265 | -10.871 |
| | BRD4(1) | wt | 55.5 ± 3.7 | 18.0 ± 1.2 | 0.912 ± 0.001 | -12.020 ± 0.033 | -1.605 | -10.415 |
| | BRD4(1) | A89V | 97.1 ± 8.5 | 10.3 ± 0.9 | 0.969 ± 0.001 | -11.140 ± 0.033 | -0.423 | -10.717 |
| | BRD4(2) | wt | 55.2 ± 4.6 | 18.1 ± 1.5 | 0.931 ± 0.001 | -5.844 ± 0.019 | 4.466 | -10.31 |
| | BRD4(2) | A420D | 22.5 ± 1.9 | 44.4 ± 3.9 | 0.990 ± 0.003 | -6.530 ± 0.035 | 3.285 | -9.815 |

*(continued on next page)*

**S2 Table** *(continued)*

| Comp | Protein | Mutant | $K_A$ ($10^6$ $M^{-1}$) | $K_D$ (nM) | N | Δ*H* (kcal/mol) | TΔ*S* (kcal/mol) * | Δ*G* (kcal/mol) |
|---|---|---|---|---|---|---|---|---|
| **PFI-1** | BRD2(1) | wt | 12.4 ± 0.4 | 80.6 ± 2.9 | 0.924 ± 0.001 | -19.230 ± 0.048 | -9.538 | -9.692 |
| | BRD2(1) | D161N | 4.90 ± 0.1 | 204.1 ± 6.6 | 0.843 ± 0.002 | -13.670 ± 0.042 | -4.609 | -9.061 |
| | BRD2(1) | D161Y | 16.6 ± 0.7 | 60.24 ± 2.6 | 1.050 ± 0.001 | -17.000 ± 0.044 | -7.201 | -9.799 |
| | BRD2(1) | Y153H | 7.4 ± 0.3 | 135.3 ± 4.9 | 1.020 ± 0.002 | -17.400 ± 0.051 | -8.039 | -9.361 |
| | BRD2(1) | E140K | 10.7 ± 0.5 | 93.5 ± 4.4 | 1.060 ± 0.002 | -14.390 ± 0.048 | -4.870 | -9.520 |
| | BRD2(1) | R100L | 9.2 ± 0.3 | 108.3 ± 3.4 | 0.950 ± 0.001 | -14.790 ± 0.036 | -5.359 | -9.431 |
| | BRD2(2) | wt | 5.0 ± 0.2 | 198.8 ± 6.6 | 0.985 ± 0.002 | -8.998 ± 0.027 | -0.008 | -8.990 |
| | BRD2(2) | Q443H | 4.7 ± 0.2 | 213.7 ± 7.6 | 0.978 ± 0.002 | -9.901 ± 0.036 | -0.936 | -8.965 |
| | BRD2(2) | R419W | 4.2 ± 0.2 | 238.7 ± 10.7 | 1.100 ± 0.003 | -6.318 ± 0.025 | 2.521 | -8.839 |
| | BRD3(2) | wt | 6.2 ± 0.2 | 161.0 ± 5.9 | 0.976 ± 0.002 | -10.540 ± 0.034 | -1.403 | -9.137 |
| | BRD3(2) | H395R | 0.70 ± 0.03 | 1386.0 ± 65.0 | 1.030 ± 0.007 | -3.552 ± 0.032 | 4.235 | -7.787 |
| | BRD4(1) | wt | 17.3 ± 0.8 | 57.8 ± 2.6 | 0.964 ± 0.002 | -19.750 ± 0.056 | -9.883 | -9.867 |
| | BRD4(1) | A89V | 8.5 ± 0.2 | 117.1 ± 2.8 | 0.918 ± 0.001 | -18.340 ± 0.038 | -8.875 | -9.465 |
| | BRD4(2) | wt | 2.8 ± 0.1 | 362.3 ± 17.1 | 1.010 ± 0.004 | -10.130 ± 0.062 | -1.464 | -8.666 |
| | BRD4(2) | A420D | 3.4 ± 0.2 | 297.6 ± 14.5 | 0.974 ± 0.004 | -10.260 ± 0.060 | -1.484 | -8.776 |

* Assay temperature = $15^0$ C, 288.15 K
