# Supplementary material for: Effect of BET Missense Mutations on Bromodomain Function, Inhibitor Binding and Stability
Source: PLoS One. 2016 Jul 12;11(7):e0159180. doi: 10.1371/journal.pone.0159180 (PMC4942050; doi:10.1371/journal.pone.0159180)
Supplement: S3 Table — (PDF) [file pone.0159180.s011.pdf]

S3 Table. Structural location and difference in melting temperature and in free energy of urea-induced unfolding of BRDs wild type and mutants

	**Position of the mutation**	**ΔTm (°C)**	**$\Delta\Delta G^{H_2O}$ (kcal/mol)**	
			**CD ($[\Theta]_{222}$)**	**Fluorescence**
**BRD2(1)**				
Y153H	αB helix	-7.0	-6.49 ± 0.64	-5.31 ± 0.82
E140K	αB helix	-10.8	-3.48 ± 0.82	-4.53 ± 0.81
R100L	ZA-loop	-5.8	-5.22 ± 0.90	-
D160N	BC-loop	-5.8	-4.10 ± 0.82	-2.33 ± 0.91
D160Y	BC-loop	-9.8	-4.02 ± 0.76	-4.20 ± 0.79
D161N	BC-loop	-1.3	-2.21 ± 0.92	-3.02 ± 0.84
D161Y	BC-loop	-4.8	-3.38 ± 0.65	-4.66 ± 0.97
**BRD4(1)**				
A89V	ZA-loop	0.1	0.99 ± 0.50	-0.79 ± 0.90
**BRD2(2)**				
Q443H	αC helix	-3.0	0.89 ± 0.50	1.68 ± 0.40
R419W	αB helix	-8.0	0.02 ± 0.48	-0.60 ± 0.38
**BRD3(2)**				
H395R	BC-loop	-2.6	2.03 ± 0.63	1.12 ± 0.54
**BRD4(2)**				
A420D	αB helix	-3.0	0.44 ± 0.48	4.93 ± 0.38
