# Supplementary material for: Effect of BET Missense Mutations on Bromodomain Function, Inhibitor Binding and Stability
Source: PLoS One. 2016 Jul 12;11(7):e0159180. doi: 10.1371/journal.pone.0159180 (PMC4942050; doi:10.1371/journal.pone.0159180)
Supplement: S4 Table — (PDF) [file pone.0159180.s012.pdf]

S4 Table. List of oligonucleotides used for site-directed mutagenesis

Mutant	Primer sequences (5’ to 3’)
**BRD2(1)**	
R100L FW	GTG GCC ATT TCT CCA GCC GGT GGA T
R100L REV	ATC CAC CGG CTG GAG AAA TGG CCA C
E140K FW	GGG CGG CCT CTA AAT GCA TGC AGG
E140K REV	CCT GCA TGC ATT TAG AGG CCG CCC
Y153H FW	GTT TAC GAA TTG TCA TAT CTA TAA CAA ACC G
Y153H REV	CGG TTT GTT ATA GAT ATG ACA ATT CGT AAA C
D160N FW	TAA CAA ACC GAC CAA TGA TAT CGT CTT GAT GG
D160N REV	CCA TCA AGA CGA TAT CAT TGG TCG GTT TGT TA
D160YFW	TAA CAA ACC GAC CTA TGA TAT CGT CTT GAT GG
D160Y REV	CCA TCA AGA CGA TAT CAT AGG TCG GTT TGT TA
D161N FW	CAA ACC GAC CGA TAA TAT CGT CTT GAT GG
D161N REV	CCA TCA AGA CGA TAT TAT CGG TCG GTT TG
D161YFW	CAA ACC GAC CGA TTA TAT CGT CTT GAT GG
D161YREV	CCA TCA AGA CGA TAT AAT CGG TCG GTT TG
**BRD4(1)**	
A89V FW	GCA GCC TGT GGA TGT CGT CAA GCT GAA CC
A89V REV	GGT TCA GCT TGA CGA CAT CCA CAG GCT GC
**BRD2(2)**	
R419W FW	GCG GCG GAT GTA TGG CTC ATG TTT TCG
R419W REV	CGA AAA CAT GAG CCA TAC ATC CGC CGC
Q443H FW	GGC ACG AAA GCT ACA CGA TGT ATT TGA G
Q443H REV	CTC AAA TAC ATC GTG TAG CTT TCG TGC C
**BRD3(2)**	
H395R FW	AAT CCC CCA GAC CGC GAG GTT GTG GCC AT
H395R REV	ATG GCC ACA ACC TCG CGG TCT GGG GGA
**BRD4(2)**	
A420D FW	CAG GAG TTT GGT GAT GAC GTC CGA TTG
A420D REV	CAA TCG GAC GTC ATC ACC AAA CTC CTG
